# Evolution of selenium utilization traits

**DOI:** 10.1186/gb-2005-6-8-r66

**Published:** 2005-07-27

**Authors:** Héctor Romero, Yan Zhang, Vadim N Gladyshev, Gustavo Salinas

**Affiliations:** 1 Laboratorio de Organización y Evolución del Genoma, Dpto. de Biología Celular y Molecular, Instituto de Biología, Facultad de Ciencias, Iguá 4225, Montevideo, CP 11400, Uruguay; 2 Escuela Universitaria de Tecnología Médica, Facultad de Medicina, Piso 3 Hospital de Clínicas, Avda. Italia s/n, Montevideo, CP 11600, Uruguay; 3 Department of Biochemistry, University of Nebraska, Lincoln, NE 68588-0664, USA; 4 Cátedra de Inmunología, Facultad de Química/Ciencias, Instituto de Higiene, Avda. A. Navarro 3051, Montevideo, CP 11600, Uruguay

## Abstract

**Background:**

The essential trace element selenium is used in a wide variety of biological processes. Selenocysteine (Sec), the 21st amino acid, is co-translationally incorporated into a restricted set of proteins. It is encoded by an UGA codon with the help of tRNA^Sec ^(SelC), Sec-specific elongation factor (SelB) and a *cis*-acting mRNA structure (SECIS element). In addition, Sec synthase (SelA) and selenophosphate synthetase (SelD) are involved in the biosynthesis of Sec on the tRNA^Sec^. Selenium is also found in the form of 2-selenouridine, a modified base present in the wobble position of certain tRNAs, whose synthesis is catalyzed by YbbB using selenophosphate as a precursor.

**Results:**

We analyzed completely sequenced genomes for occurrence of the *selA*, *B*, *C*, *D *and *ybbB *genes. We found that *selB *and *selC *are gene signatures for the Sec-decoding trait. However, *selD *is also present in organisms that do not utilize Sec, and shows association with either *selA*, *B*, *C *and/or *ybbB*. Thus, *selD *defines the overall selenium utilization. A global species map of Sec-decoding and 2-selenouridine synthesis traits is provided based on the presence/absence pattern of selenium-utilization genes. The phylogenies of these genes were inferred and compared to organismal phylogenies, which identified horizontal gene transfer (HGT) events involving both traits.

**Conclusion:**

These results provide evidence for the ancient origin of these traits, their independent maintenance, and a highly dynamic evolutionary process that can be explained as the result of speciation, differential gene loss and HGT. The latter demonstrated that the loss of these traits is not irreversible as previously thought.

## Abstract 2

Completely sequenced genomes were analyzed for occurrence of *SelA*, *B*, *C*, *D *and *ybbB *genes. *SelB *and *SelC *were found to be signatures for the Sec decoding trait, while *SelD *defines the overall selenium utilization.

## Background

Selenium (Se) is an essential trace element for numerous organisms that belong to the three domains of life. The most relevant biological form of selenium is the rare amino acid selenocysteine (Sec), the selenium analog of cysteine (Cys). Sec is co-translationally incorporated into protein [1-3]. In functionally characterized selenoproteins, Sec is the catalytic group in the active site and is directly involved in redox catalysis. It is thought that Sec confers a functional advantage over cysteine at these active sites, increasing the catalytic efficiency of the enzymes [4]. Despite this selective advantage, the set of selenoproteins in any given organism is small [5,6].

Sec is inserted into selenoproteins at in-frame UGA codons (usually termination codons) by tRNA^Sec ^(SelC) [2,7]. Interpretation of UGA as Sec requires translational reprogramming, which is provided by the Sec insertion sequence (SECIS) element, a *cis*-acting stem-loop structure present in the selenoprotein mRNA [2]. The decoding of Sec in bacteria also involves a Sec-specific elongation factor (SelB) which binds GTP, the SECIS element and the tRNA^Sec ^[8,9]. In eukaryotes, this function is carried out by two proteins: EF-Sec and SECIS-binding protein (SBP2). EF-Sec is a Sec-specific elongation factor, distantly related to bacterial SelB, that binds GTP, tRNA^Sec ^and SBP2; this latter protein, in turn, binds the SECIS element [10]. Sec synthesis is the other part of the metabolic pathway required for biosynthesis of selenoproteins. It takes place on tRNA^Sec^, which is first aminoacylated with serine (by a canonical seryl-tRNA synthetase) and then modified to selenocysteinyl-tRNA, in the reaction that uses selenophosphate as the selenium donor [9]. In the Bacteria, this reaction is catalyzed by Sec synthase (SelA). The functional equivalent of SelA in Archaea and Eukarya has not been described. A phosphoseryl-tRNA^Sec ^kinase (PSTK) has been recently identified only in eukaryotic and archaeal Sec-incorporating organisms [11]. It has been suggested that this protein can play a role in Sec biosynthesis and/or regulation. The synthesis of selenophosphate is catalyzed by selenophosphate synthetase (SelD) from ATP and selenide in both prokaryotes and eukaryotes.

Selenophosphate has also been described as a precursor for the last step of the synthesis of the modified base 5-methylaminomethyl-2-selenouridine in the wobble position of the anticodons of Lys, Glu and Gln tRNAs [12], and this reaction was reported to be catalyzed by YbbB in *Escherichia coli *[13]. The function of this modified base is not known.

Thus, considerable efforts in recent years have been made to elucidate molecular details of Sec decoding in the three domains of life. In addition, the selenoproteome of several species has been the subject of intensive research [5,6,14-16]. Despite this progress, fundamental issues relating to the evolution of Sec utilization remain unclear. On the basis of the complexity and similarity of the Sec-insertion mechanisms in different organisms, it has been proposed that the Sec-decoding trait arose once, before the division of the three domains of life, and was subsequently lost in some lineages. It is also thought that once an organism has lost the Sec-insertion system, it cannot re-emerge. Whether the Sec biosynthesis and insertion pathway evolved before the fixation of the genetic code or whether this was a late addition is not known [17-19].

Here we provide a map of Sec-incorporating and selenouridine-utilizing organisms within the tree of life, based on the analysis of completely sequenced genomes. From phylogenetic analysis of all components of the Sec-decoding machinery, we present clear evidence for the loss of the trait in many lineages at different taxonomic levels, and examples of acquisition of the trait by horizontal gene transfer (HGT). In addition, we describe and explain the maintenance of selenophosphate synthetase in non-Sec-incorporating organisms, and use this information to define a selenouridine-utilization trait as well as a general selenium-utilization trait. We find that the 2-selenouridine pathway can also be acquired by HGT. These data suggest that the loss of selenium utilization is not irreversible.

## Results

### A map of selenium utilization within the tree of life

**Figure 1:**
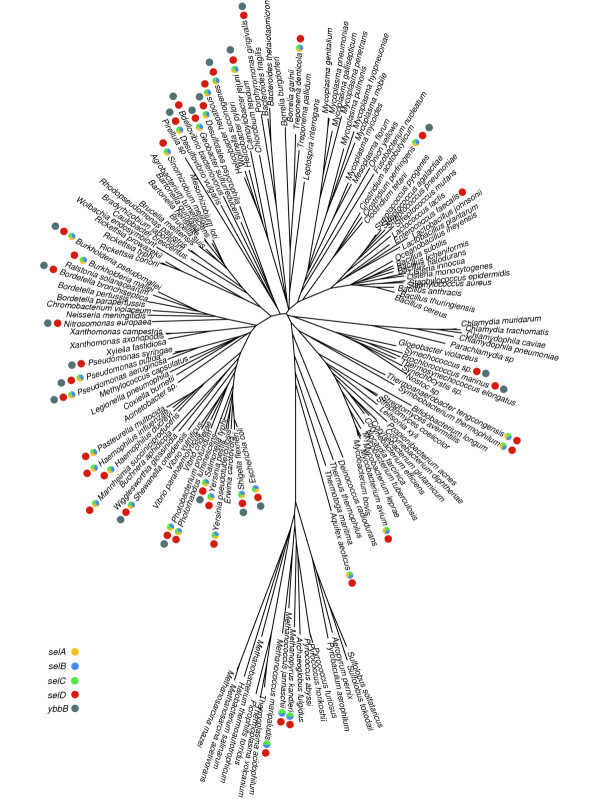
Distribution of selenium-utilization traits. The figure depicts the species tree for all organisms completely sequenced so far, based on the phylogenetic reconstruction using the small subunit rRNA sequences and is in good agreement with other consensus phylogenetic trees. Species able to decode Sec are those possessing *selA *(yellow) (excluding Archaea), *selB *(blue), *selC *(green) and *selD *(red). The presence of *ybbB *(gray) and *selD *indicates the ability to synthesize 2-selenouridine.

Figure 1 displays a phylogenetic tree, based on rRNA, of the 155 species whose entire genomes have been sequenced (see Materials and methods for the rationale behind the use of rRNA and other alternatives). The criteria for the occurrence of the Sec-decoding trait included the presence of known genes involved in Sec decoding (that is, *selA*, *selB*, *selC*, *selD*), and at least one gene encoding a known selenoprotein in the genome, inferred by the presence of a UGA codon within a coding region (at the location corresponding to Cys in homologs) followed by a downstream SECIS element. Using these criteria, a total of 29 bacterial and three archaeal species were found to be Sec decoding. These criteria were in agreement with experimental evidence when available. A map of *selA*, *selB*, *selC *and *selD *within the species tree is provided in Figure 1.

Despite the bias among the prokaryotic genomes so far sequenced [20], in which proteobacteria are over-represented and some phyla are not represented at all, the taxa distribution of Sec incorporation revealed interesting features of this trait. First, the trait is widely distributed and present in numerous bacterial phyla (Proteobacteria, Firmicutes, Spirochaetes, Actinobacteria, Aquificae). Second, we observed the presence and absence of the trait in taxa within monophyletic groups. This phenomenon takes place within clades at different evolutionary levels, namely phylum, class, order, family, genera, and even species and is illustrated in Figure 1. Considering the genus level, we observed this phenomenon within *Pseudomonas*, *Treponema*, *Clostridium *and *Yersinia*. The more revealing case of this absence/presence pattern is that of the KIM, biovar Mediaevalis and CO92 strains of *Yersinia pestis*. Whereas the KIM strain possesses a functional Sec-decoding machinery, the CO92 and Mediaevalis strains carry a confirmed sequenced *selB *pseudogene, whose coding region is disrupted by a frameshift [21]. Furthermore, the CO92 and the Mediaevalis strains possess *selA*, *selC *and *selD*, indicating that the loss of the ability to decode Sec is very recent. Moreover, at position 203 of the α-subunit of formate dehydrogenase type O there is a UGA codon in the three strains, which is decoded as Sec by the KIM strain, but could not be decoded as such in the CO92 and the Mediaevalis strains. The three strains also possess formate dehydrogenase type H with a Cys-containing α-subunit (fdhH).

*SelB *and *selC *can be considered as the gene signature of organisms able to decode Sec: their presence in genomes always coincides with that of *selA *(excluding archaeal and eukaryote domains), *selD*, and selenoproteins. A putative ortholog of *selA *is present in *Helicobacter pylori *(a Sec-non-incorporating organism). The presence of this protein in the two strains of this species is intriguing and raises the question of whether this protein serves a different function or is just a remnant of the Sec-decoding machinery. The case of *selD *is different, because it is present in several species that lack other genes necessary for Sec decoding. *SelD *orthologs are indicated in red in Figure 1.

**Figure 2:**
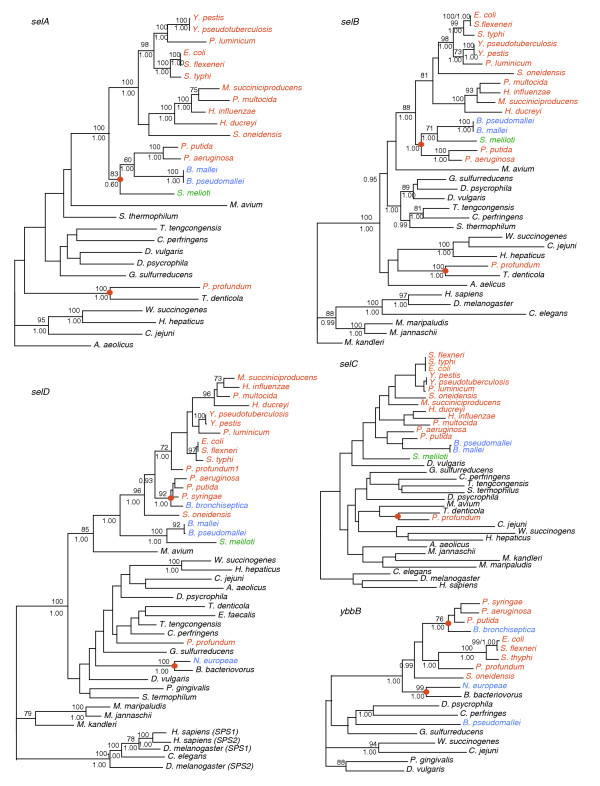
Phylograms of *selA*, *selB*, *selC*, *selD *and *ybbB*. Phylograms were inferred using phyML2.4 from curated T-Coffee alignments. The values above and below each branch indicate boostrap values (>70) of maximum likelihood analysis and posterior probabilities (>0.90) of Bayesian analysis respectively. In all trees γ-proteobacteria are highlighted in red, β-proteobacteria in blue and *Sinorhizobium meliloti *(α-proteobacteria) in green. Red circles denote putative horizontal gene transfer events.

Bacteria possessing *selD *but not the Sec-decoding trait include *Bordetella bronchiseptica*, *Pseudomonas syringae*, *Porphyromonas gingivalis*, *Nitrosomonas europaea*, *Bdellovibrio bacteriovorus *and *Enterococcus faecalis*. In addition, two cyanobacteria - *Prochlorococcus marinus *and *Synechococcus *species - possess a putative *selD *homolog with a 320-amino-acid amino-terminal extension with similarity to NADH dehydrogenases. All *selD*s from non-Sec-incorporating bacteria, excluding those of cyanobacteria, are likely to be 'true orthologs' to *selD *from Sec-incorporating bacteria because the topology of the *selD *phylogeny parallels the topology of species for both Sec-incorporating and non-Sec-incorporating species (Figure 2d), and because the sequence signatures of bacterial *selD *are present and are of similar length (see Additional data files). Although it is difficult to sketch an evolutionary history for the *selD *from cyanobacteria, it is clear that these proteins have many features of selDs and could be viewed as true selenophosphate synthetases (see below).

The fact that selenophosphate is also the precursor for the synthesis of 2-selenouridine [12], a modified base that is present at the wobble position of Lys, Glu and Gln tRNAs, suggests that *selD *may have been maintained in these organisms to generate selenophosphate for 2-selenouridine synthesis. Thus, we investigated the distribution of *ybbB*, a gene encoding the catalyst of the last step of 2-selenouridine synthesis [13], and its association with *selD*.

**Figure 3:**
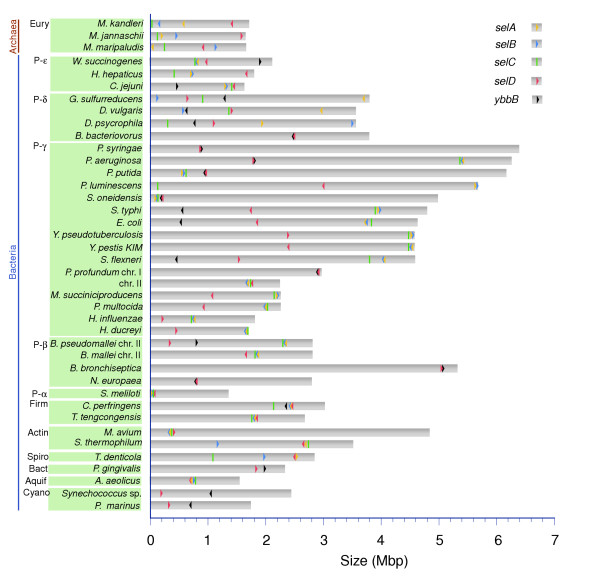
Genome location of *selA*, *selB*, *selC*, *selD *and *ybbB*. Each bar represents one replicon of a species. On the vertical axis the species name, phylum, and domain are specified. The horizontal axis corresponds to the replicon size. Location of *selA *(yellow), *selB *(blue), *selC *(green), *selD *(red) and *ybbB *(black) is indicated; arrows denote direction of transcription.

A search across genomes for *ybbB *(indicated in gray in Figure 1) revealed that six out of seven *selD*-containing and non-Sec-decoding species also contained *ybbB*. In addition, all *ybbB*-containing organisms also possess *selD*, including cyanobacteria. Furthermore, in most of these species, except *P. gingivalis *and cyanobacteria, both genes are located contiguously and arranged in an operon (Figure 3), as has been previously suggested [13]. This gene organization is also seen in some species that incorporate Sec and possess *ybbB*: *selD *is contiguous to *ybbB *in some genomes, but is rarely contiguous to the *selA*-*selB *operon (Figure 3).

The analysis of *selA*, *selB*, *selC*, *selD *and *ybbB *genes also revealed that, within the set of species that incorporate Sec, many, but not all, organisms, possess *ybbB *and vice versa. In other words, the set of species that incorporate Sec into protein overlaps with, but is different from, the set of species that possess *ybbB *(Figure 1). It is important to note that a low-identity homolog to bacterial *ybbB *is present in *Methanococcus jannaschii *and *Methanopyrus kandleri*, and absent in other archaea, suggesting that this base modification might not be unique to bacteria.

Finally, we investigated the presence of additional genes linked to the selenouridine synthesis trait by searching genomes for genes that occur in organisms possessing *ybbB *and are absent in organisms lacking *ybbB*. This search did not identify any additional gene associated with this trait. Thus, the overall analysis allows us to corroborate that the two products of these genes form a pathway with 2-selenouridine in the tRNA as the final product. However, only *ybbB *is the gene signature of this trait. On the other hand, the dual use of selenophosphate (for Sec decoding and 2-selenouridine biosynthesis) makes *selD *a signature of a broader trait of selenium utilization, and our data suggest that both Sec decoding and selenouridine traits are independently maintained, but both require *selD*.

### Phylogeny of *selA*, *selB*, *selC*, *selD *and *ybbB*: evidence of horizontal gene transfer (HGT) of Sec-decoding and selenouridine synthesis traits

The phylogenies of *selA*, *selB*, *selC*, *selD *and *ybbB *shown in Figure 2 are neither mutually coherent nor match the 'species tree' (Figure 1). This does not necessarily imply an error in the phylogenetic reconstruction since the evolutionary history of each gene could be different. Many nodes are mutually consistent across different methods and have high statistical support. Certain anomalous situations occur with distantly related organisms (deep nodes), which could be due to the limitations of these analyses. However, some of the inconsistencies may be considered as 'genuine' and raise HGT as the most likely alternative explanation.

A striking observation is the clustering of *P. profundum *(a γ-proteobacterium) with *T. denticola *(a spirochete) at a basal position of the *selA*, *selB *and *selC *trees. This topology is consistent in various phylogenetic reconstruction methods and has high statistical support in all cases. The congruence of the trees sustains the idea that these genes were horizontally transferred to *P. profundum*. Several facts provide further support for this proposition. The *P. profundum *genome encodes four selenoproteins: two glycine reductases A, one glycine reductase B and selenophosphate synthetase. This selenoproteome is entirely distinct from that of γ-proteobacteria and very similar to that of *T. denticola*, which consists of glycine reductase A, two glycine reductases B, selenophosphate synthetase, glutathione peroxidase and thioredoxin. Furthermore, glycine reductase is absent in every other proteobacterial genome. In addition, *P. profundum *is the single prokaryotic genome that has two *selD*s: one encodes a Sec-containing isoform that is located next to the *selAB *operon, on chromosome II; the second encodes a Cys-containing enzyme that is adjacent to *ybbB *on chromosome I. The phylogeny of *selD *places the Cys isoform within the γ-proteobacterial clade as expected according to the organismal phylogeny, whereas the Sec isoform does not cluster with *T. denticola *or with γ-proteobacteria. Altogether, these results indicate that it is highly unlikely that *P. profundum *has acquired the Sec-decoding trait by vertical descent, raising HGT as the obvious alternative. In addition, we analyzed the codon usage of *selA *and *selB*, looking for an anomalous pattern, using the method described by García-Vallvé [22]. These genes do not display biased values of codon usage with respect to the rest of the genes. This result could indicate that *P. profundum *has already adapted the codon usage of these genes to its internal values. A recent paper suggested that the compatible codon usage between foreign genes and recipient genomes increases the probability of HGT [23]. Since *T. denticola *and *P. profundum *do not share the same environment, it is likely that the HGT took place from a species of spirochete, a bacterial phylum exhibiting great variability in habitat and physiology [24].

An additional incongruence is observed when the *selA*, *selB*, *selC *and the species trees are compared among *Pseudomomas *spp. (γ-proteobacteria), *Sinorhizobium meliloti *(α-proteobacterium) and *Burkholderia *spp. (β-proteobacteria) (Figure 2). The evolutionary history of these genes is, however, difficult to solve.

Conflicts relating to the selenouridine synthesis trait were also observed. The consistent and statistically supported cluster between *Bordetella bronchiseptica *(a β-proteobacterium) and *Pseudomonas *spp., within the γ-proteobacteria clade in both *selD *and *ybbB *gene trees, strongly suggests an event of HGT from *Pseudomonas *spp. to *B. bronchiseptica*. A situation that cannot be explained by vertical descent is also the cluster of *Nitrosomonas europae *(a β-proteobacterium) and *Bdellovibrio bacteriovorus *(δ-proteobacteria) in *selD *and *ybbB *phylogenies (Figure 2). The location of *ybbB *and *selD *genes also supports this possibility: while arranged in an operon in *N. europeae *and *B. bacteriovorus*; they are distant in the genomes of the other δ-proteobacteria (Figure 3). Furthermore, *B. bacteriovorus *is a predatory bacterium with a multiplication phase within many Gram-negative bacteria [25]. Thus, the ready access to the prey's genetic information and vice versa might be a possible explanation for this HGT event.

## Discussion

The distribution of the Sec-decoding trait within the 'species tree' prompts the question of how it evolved. A supported conclusion from our data is the common origin of *selA*, *selB*, *selC *and *selD *in the domain Bacteria. This is based on the absence of close paralogs for Sec-decoding genes in bacteria, the high bootstrap value for the bacterial node in all phylogenies, and the presence of bacterial sequence signatures in *selA*, *selB*, *selC *and *selD *sequences (see Additional data files). The phylogenies of *selB*, *selC *and *selD *also indicate that the archaeal and eukaryal Sec-decoding genes cluster together. This is further supported by the similar overall organization of the Sec-decoding machinery in Archaea and Eukarya [26-28]. The emergence of the Sec-decoding trait before the division of the three domains has been previously postulated [18,29]. The evolution of the Sec insertion system only once is certainly the most parsimonious evolutionary scenario. However, this does not necessarily imply that every gene involved in Sec-decoding has a common origin. This is exemplified by *selA*: no clear ortholog has been found in Archaea and Eukarya. This suggests that the mechanism of Sec biosynthesis and insertion could have been adjusted during evolution.

Assuming the common origin of the Sec-decoding trait, it is possible to sketch a scenario compatible with our results in order to explain the pattern of presence/absence of the Sec-decoding trait. We propose that this pattern is the result of two mechanisms, primarily speciation and differential gene loss, with some contribution from HGT. Regarding the selenouridine synthesis trait, the results also suggest a common origin in the bacterial domain, as well as the possibility that 2-selenouridine pathway can be acquired by HGT.

An important issue in the evolution of Se utilization traits relates to the selective forces operating to maintain, loose or acquire the traits. Although it is not possible to draw conclusions, the search for a common biochemical, physiological or ecological trait in organisms possessing/lacking either or both traits provides interesting clues. The analysis of the prokaryotic selenoproteome revealed that formate dehydrogenase is present in most organisms capable of Sec decoding, exceptions being *T. denticola*, *P. profundum*, *Clostridium perfringens *and *Thermoanerobacter tengcongensis *[6]. Formate dehydrogenase plays a key role in anaerobic respiration. Indeed, most of these species are obligatory anaerobes or facultative aerobes; the sole exception was *S. meliloti*, a symbiotic nitrogen-fixing obligatory aerobe that lives in the oxygen-limited environment of the nodule [30]. Formate dehydrogenase is the single Sec-containing polypeptide encoded in the *Sinorhizobium meliloti *genome [6,30], suggesting that the presence of the trait may be important for respiration under conditions of restricted oxygen supply. On the other hand, glycine reductase is present in *T. denticola*, *P. profundum *and *T. tengcongensis *and several species of the genera *Clostridium *except *C. perfringens*. Glycine reductase is an enzymatic complex that allows certain anaerobic bacteria to conserve energy via a soluble substrate level phosphorylation system [31]. Sec is more reactive than Cys by virtue of the lower pKa and higher nucleophilicity of selenol group compared to that of the thiol group [12], and can increase the pH range at which certain enzymes are active [32]. This might have conferred a selective advantage improving catalytic efficiency of proteins.

Regarding selective forces operating on the evolution of the selenouridine synthesis trait, we begin from the fact that synthesis of 2-selenouridine is carried out exclusively at the wobble position (first of the anticodon) of the tRNAs for lysine, glutamate and glutamine (the only amino acids encoded by twofold purine-ending codons). Several modifications of this base have been reported to be essential for correct decoding; thiouridine, in particular, would convert the base into an ionized form that would favor pairing with A and G, and avoid pairing with U or C, contributing to the discrimination of twofold codons ending in purine from those ending in pyrimidine [33]. The low pKa value of 2-selenouridine of these tRNAs would be consistent with this argument and it has been suggested that this would also favor base-pairing with G [34]. Thus, we postulate that selenium modification of tRNAs matching twofold codons might be a refinement in the base discrimination at the wobble position. The interaction of the first base of the anticodon with the third base of the codon plays an important role in the efficiency and accuracy of the translation process, suggesting that this base modification could be linked to certain aspects of codon usage. In any case, it should be stressed that ybbB null *E. coli *has no apparent phenotypic differences to wild type-*E. coli *and does not alter nonsense suppression phenotype [13].

One of the driving forces for the loss of the traits probably relates to the variability of selenium abundance in the environment. The absolute dependence of organisms on Se can compromise their existence if dietary Se becomes limiting. In these situations, enzymes containing Sec as catalytic residues could have evolved into Cys-containing proteins or, alternatively, both Sec-containing and Cys-containing forms could be maintained. This latter case is exemplified by the genome of *M. maripaludis*, which encodes several Sec-containing proteins and also homologs that contain cysteine in place of Sec. In a medium that contains adequate amounts of selenium, this organism represses the synthesis of the cysteine homologs, but this repression is not observed in a mutant with disrupted *selB *[35], suggesting that the cysteine homologs are a backup system in case of selenium scarcity. Nevertheless, the existence of organisms carrying only one of the selenium-utilization traits suggests that selenium availability might not be the sole factor involved in the loss of either trait. It is also possible that the higher reactivity of selenium over sulfur in biological molecules might have had a role in counterselecting the pervasive use of Sec and/or selenouridine in living systems.

## Conclusion

This paper provides an organismal map for Sec-decoding and 2-selenouridine synthesis traits within the tree of life, and defines *selB *and *selC *as the gene signature of the Sec-decoding trait, *ybbB *as the gene signature of selenouridine synthesis, with *selD *defining overall selenium utilization. We show that the set of species that incorporate Sec overlaps with, yet is distinct from, the set of species that synthesize 2-selenouridine, and our data suggest that Sec decoding and 2-selenouridine traits can be independently maintained, and both require *selD*.

Analysis of the phylogenies of the Sec-decoding and 2-selenouridine synthesis genes provides evidence for the ancient origin of these traits and demonstrates that their evolution is a highly dynamic process that occurs at different evolutionary levels, namely phylum, class, order, family, genera, and even species. We show that this process can be explained as the result of speciation and differential gene loss, and provide conclusive evidence that the loss of these traits is not irreversible as previously thought, and that entire sets of genes can be acquired by HGT. It is striking that the genetic code of an organism and the amino-acid repertoire can be 'laterally' expanded.

The study of selenium-utilization traits, which directly associate protein synthesis with a discrete set of genes, can contribute to the understanding of basic questions regarding the evolution of the genetic code and the translation machinery.

## Materials and methods

### Sequences of *selA*, *selB*, *selC*, *selD *and *ybbB*

Complete genome sequences of 194 prokaryotes were retrieved from GenBank [36] as of 20 October 2004, representing 151 species.

Annotated sequences corresponding to *selA*, *selB*, *selD *and *ybbB *prokaryotic genes were retrieved from GenBank, and used as queries to perform local BLAST searches across a database generated with the 194 genomes. For *selA*, *selD *and *ybbB*, hits with an e-value below e^-15 ^were recovered; for *selB*, the cutoff e-value was e^-30 ^to decrease the number of hits corresponding to other translation factors. A total of 242 *selB*, 48 *selA*, 47 *selD *and 25 *ybbB *sequences were recovered. The sequences were aligned using ClustalW [37], a raw phylogenetic analysis was conducted and clear nonorthologous sequences were discarded. This dataset was manually curated, and the number of sequences was reduced to 29 *selA*, 32 *selB*, 41 *selD*, and 21 *ybbB *sequences. These datasets were used as queries for BLAST searches but no new sequences were identified. Finally, only one sequence by strain was included and the set was supplemented with sequences of three representative eukaryotes (*Caenorhabditis elegans*, *Drosophila melanogaster *and *Homo sapiens*).

Most sequences of *selC *were retrieved from GenBank or identified using tRNAscan software with default parameters [38,39]. The sequences of *Wolinella succinogenes*, *Helicobacter hepaticus*, *Burkholderia mallei *and *Thermoanaerobacter tencongensis*, were found changing the parameters to 'Cove-only search mode' and lowering the tRNA Cove cutoff score to 6.

All sequences are provided aligned in the Additional data files.

### Alignments and phylogenetic reconstruction of gene trees

Several phylogenetic gene trees were built using different inference methods performed on different sequence alignments. Sequences were aligned with T-coffee version 1.37 [40], ClustalW 1.8 and Dialign-2 [41] using different parameters. The 'score' option of the T-coffee software was enabled to assess alignment quality. The alignments were then visually inspected, compared and uncertain sites were removed. In another approach, we applied the g-blocks software [42] to remove unstable blocks with 2 different sets of parameters. The final alignment sets were the following: i) raw alignments using each software with two different sets of parameters ii) 'sub-alignments' obtained removing the unstable blocks from the raw alignments using g-blocks software, and iii) 'sub-alignment' obtained using the '-score' option of T-coffee for evaluation of the alignment, then the low scoring regions were removed manually.

For each of these alignments, we applied several phylogenetic reconstruction methods including Neighbor Joining using MEGA software [43], Maximum Likelihood (ML) using phyML 2.4 software [44] and Bayesian approaches using MrBayes 3.0b4 [45]. For each of these methods, different transition matrices (WAG and JTT) and evolutionary models were tested. In total, more than 80 trees were analyzed for each gene. The gene trees presented in Figure 2 were built using the T-coffee alignment evaluated with the '-score' option and manually refined. The ML and Bayesian trees were built using WAG matrix and gamma+invar model of evolutionary change. In the ML method, the assessment of node reliability was done using 100 bootstrap replicates. In the case of Bayesian analyses, four heated Markov chains were started from random trees and run for 1,000,000 generations each. Chains were sampled every 500 generations to assure independence. Sample points prior to reach stationary (200) were discarded as 'burn-in'.

Almost all trees yielded similar topologies and, more important, all of them supported the conclusions. In particular, the HGT results were reproduced with any of the alignments and phylogenetic trees.

### Species tree

Different species trees were initially constructed, based on small-subunit (SSU) rRNA, EF-Tu/EF-1α (a highly conserved translation elongation factor present in all organisms), and a concatenated set of 9 ortholog sequences present in all prokaryotes. A set of aligned SSU-rRNA sequences was retrieved from the Ribosomal Database Project (RDP) [46], release 2.1, missing sequences were retrieved from Genbank and aligned against the set from the RDP using the profile option of ClustalW.

EF-Tu and EF-1α were recovered using a similar approach to that described for *selA*, *selB*, *selD *and *ybbB*, and aligned using T-coffee. An all-against-all BLAST search was performed sequentially and best reciprocal hits were identified as putative orthologs. A set of nine genes was obtained. These sequences were aligned with ClustalW and concatenated. In all cases we used the neighbor-joining (NJ) algorithm to build the trees from different distance matrices using MEGA software [43]. In the case of SSU-rRNA, the Tamura-Nei (TN93) distance with pairwise-deletion was calculated. For the amino-acid alignments we use the JTT transition matrix.

The different 'species trees' display some discrepancies. In any case, the conclusions drawn are maintained with any of the above mentioned 'species tree'. The SSU-rRNA was finally adopted because it is by large the commonest used, and trees inferred for this gene are sound descriptors of the general evolutionary history of prokaryotes. This tree also recovers the major groups described in *Bergey's Manual *[47].

### Codon bias analyses

Codon bias was evaluated according to the method described in [22]. This method uses the Mahalanobis distance measure for detecting outliers in a multivariate distribution.

### Search for additional genes linked to Se-U trait

To study the possible association of a certain gene with *ybbB *we run an all-against-all BLAST search with an e-value threshold of e^-10 ^among organisms carrying *ybbB*, to pick up homologs present in all these genomes. Then we used this set of genes to run a new BLAST search against a control set of closely related species lacking a *ybbB *homolog. This search detected no gene. When we excluded Sec-decoding species from the control set, we were able to recover a single gene: *selD*.

## Additional data files

Additional data are available with the online version of this paper. Additional data file 1 is a table containing the gene locations of *selA*, *selB*, *selC selD *and *ybbB *in the genomes analyzed in this work. Additional data file 2 contains the sequence alignments of *selA*, *selB*, *selC selD *and *ybbB *of the genomes analyzed in this work.

## Supplementary Material

Additional data file 1 A table containing the gene location of *selA*, *selB*, *selC selD *and *ybbB *in the genomes analyzed in this work Click here for file

Additional data file 2 Sequence alignments of *selA*, *selB*, *selC selD *and *ybbB *of the genomes analyzed in this work. Click here for file
