# Supplementary material for: Evolution of selenium utilization traits
Source: Genome Biol. 2005 Jul 27;6(8):R66. doi: 10.1186/gb-2005-6-8-r66 (PMC1273633; doi:10.1186/gb-2005-6-8-r66)
Supplement: Additional data file 2 — Sequence alignments of selA, selB, selC selD and ybbB of the genomes analyzed in this work. [file gb-2005-6-8-r66-S2.doc]

**SELA**

CLUSTAL FORMAT for T-COFFEE Version_1.37, CPU=0.00 sec, SCORE=53, Nseq=29, Len=510

Helhep --------VQHLLKSLPKVDTLLAHNEL----K----MFQKSTLLPLIQSHLSLLREN-I

Camjej ---------MNKFRTFPQINTLIEDES--------LKSYPFYIKAFFCKKVVAKLKENFF

Wolsuc --------MSHPLRSLPKIDKILQESRF----AD----HSKELLTTLARDYLEEIRAQ-F

Aquaeo --------MKSLLRQIPQISKVVEI---------FKKKYPEIYVVKAAREVAEKYRKE-I

Cloper -------MTKELLRALPKIDEILGIFN-----EDFLNENGRETIVSALRDIINENRKA-I

Despsy ------MPKNNLLRTLPKVDESIAQLR-----STGVAYSSDFVLKKSVQTCIARERQGIL

Mycavi ------------------------------------------------------------

Pseaer ----------MSSVRLPSVDRLLRSAAA----APLHQRYGREALLATLRDLLDELREP-A

Pseput ----MSNSLASDTPRLPSIDTLLRHPAC----LPLIDRHGRDAVLNTLRQLLDDLREP-A

Burpse MSGPGASELNALLARAPSVERVLSSEEM----QPLVDEYGRTRALAAVRASLAAWRDA-A

Burmal MSGPGASELNALLARAPSVERVLSSEEM----QPLVDEYGRTRALAAVRASLAAWRDA-A

Sinmel ------MSGPVDLRALPSVDQMLNAAAV----SPLVEQHGRAVVTDELRKVLGEVRLA-V

Haeinf --------MTALFQQLPSVDKILKTPQG----LQLITEFGHTAVVATCRELLTQARQF-I

Mansuc --------MTALFQQLPSVDKILKTPQG----EQLVTEFGHSAVVNCCRHLLAQAREK-I

Pasmul ---------MSLYSSLPSIDKLLKTPEG----ARLSHEFGHTAVVNICRQLIEQGREY-I

Haeduc --------MQKLLSVIPSVDKLIKQLAG----IALIQQYGHQAFVAQARQVIDSLRQH-I

Yerpes --VTMSAEPHPLYRQLPAIDRLLNEPEM----APLLAEYGPVLLADTLRQLQAEAREY-I

Yerpse ----MSAEPHPLYRQLPAIDRLLNEPEM----APLLAEYGPVLLADTLRQLQAEAREY-I

Saltyp ----MTSETRTLYSQLPAIDRLLHDSAF----LSLRDRYGHTQVVDLLRRMLDDARDV-I

Shifle ----MTTETRSLYSQLPAIDRLLRDSSF----LSLRDTYGHTRVVELLRQMLDEAREV-I

Esccol ----MTTETRSLYSQLPAIDRLLRDSSF----LSLRDTYGHTRVVELLRQMLDEAREV-I

Pholum ----MTHKPCLLYSQLPAIDRLLREPQI----TPLVEQYGQTLVTATLRRMQEQARIN-I

Sheone MMTQVPDTPQALYRALPSMDSLLADAVL----APLLQRYGKAAVKAALDSQLRTAREL-I

Desvul --------VNRLFRALPSVDALLTALTTPTHGHDTFADLPRALLRDLVNDFLDARRED-I

Geosul ---------VTIFSRIPKVDRILEQDEV----RRLLADHPRPTVLAAVRTALDALRAE-A

Symthe ----MKPWQQSALRAIPPVNEILETPTG----QRLLAERARALVVDAVGEALDALRRK-I

Theten --------MEDLYRKLPSVDEILREEKI----NEVLKFNKREVVKNCIREVLERYREK-I

Treden ----MAKENTNPLARIPQVEKLLNEGIL----KNTAALIGRPFVVKKASEYLEDIRKK-A

Phopro -------MTINALSAIPQIGKMLEQAFL----SPYIERLSQPVVADLVREQVNGYKKDHL

Helhep CSNTLSPK--ELESALNEIIPVIKKKAIEATKPTLTRVINATGVVIHTNLGRSVLSPQIL

Camjej QDE----------ISKDKLLLEIKKEIKTFYRKDLQSVINASGVVIHTNLGRSVIHEELY

Wolsuc LQDATP------IPPSESILQEVERRYEASLAPSLVPLVNATGIIVHTNLGRSVFAPELI

Aquaeo IEGKR--------KDLNGFLEDVERKIKSLMKPNIKRVINATGVVINTNLGRAPLSKDVI

Cloper LNEEVD-----YALTKEEAKSKCEHRLLKKRERNLKRVINGTGVVIHTNLGRSLLSKEAT

Despsy AGKITEK------RAEDSWHACFLKELKERQAPNLRRMINGTGVVIHTNLGRSLLSSQVA

Mycavi ----------------------------------LRPVINATGVVVHTNLGRAALDAVV-

Pseaer RHGALAE----IELSEAVLAGRAGERLAAQHASRVRRVFNLTGTVLHTNLGRALLPDEAI

Pseput RNGELGSA----ELAPEILLGRSGERLALQQRSQVRRVFNLTGTVLHTNLGRALLPDEAI

Burpse RRDPAAAG---T-PDDARIAADVRARLAADAAAAMRAVFNLTGTVLHTNLGRALLPDDAV

Burmal RRDPAAAG---T-PDDARIAADVRARLAADAAAAMRAVFNLTGTVLHTNLGRALLPDDAV

Sinmel RSGGALPG-------KDGIVAALLSRLDDRSRSNLRPLFNLTGTVLHTNLGRALLAQEAV

Haeinf KKNNQLPE---YFSNFDRTFLEIHSHLQKQNQVQIKAVHNLTGTVLHTNLGRALWSEAAQ

Mansuc KIEKKLPH---FFTDFNHTIAEVNRYLANQQQVKIKSVHNLTGTVLHTNLGRALWAQSAQ

Pasmul KNENKLLP---VFQDISDTLREIELQLHAQSQVKIQSVHNLTGTILHTNLGRALWSDAAQ

Haeduc LTHQSLP----VDQPADNLLSLIATNLQQTQQIAMKKVFNLTGTILHTNLGRAIWSEAAI

Yerpes GQFHTLAD---WC---ADWPAALRQRL-NQRQPALKPVFNLTGTVLHTNLGRAPLAESAI

Yerpse GQFHTLAD---WC---ADWPAALRQRL-NQRQPALKPVFNLTGTVLHTNLGRAPLAESAI

Saltyp RNTQTLPD---WY---ADWAQEAKLRLENAAQSALRPVINLTGTVLHTNLGRALQAQEAI

Shifle RGSQTLPA---WC---ENWAQEVDARLTKEAQSALRPVINLTGTVLHTNLGRALQAEAAV

Esccol RGSQTLPA---WC---ENWAQEVDARLTKEAQSALRPVINLTGTVLHTNLGRALQAEAAV

Pholum KQYQALPD---WC---DNWASALGQQLEQKQALALKPVFNLSGTVIHTNLGRALMAESAI

Sheone AQERRLPA---WCANPSLLNGYLVDQLSKDYSHSLKPVWNLTGTILHTNLGRAQQSEAAI

Desvul RQGRIDAP---ESLAAERLLPSLVQSVGRRSRAHFRRVLNGTGVVIHTNLGRSLLAPAAT

Geosul RAGSLREQ----DLADGAVAGRVGREVARSTACSLRRVVNGTGVVIHTNLGRSPLAERVR

Symthe AAAASEEELAVLDRSPEGLAADAAARLSRRLRPRLRRVINATGVVIHTNMGRAPLAEEAV

Theten RRGEVKK------IDIEKILEDVVSQIEEKKKMSLRRVVNGTGIILHTNLGRALFPPQVK

Treden RAGGDVP-------LLKACAEEICKLCRPIIRTKITPVINATGIILHTNLGRSPLPENIW

Phopro RNPEHIP------FSLETLYSRIERACWQVNQQRLQRVINGTGVIVHTNLGRSPLDADVW

. : * :* :::**:**:

Helhep DEITPFLRSYHTLEYDLAKGKRSERYT-HTKQILCEMFGCEDALLVNNNAAAVLLILNTF

Camjej EACKDIICNYSNVEFDLENGKRGSRYA-LVLEKLKMLFECEDALVVNNNAAAVFLVLNSL

Wolsuc EEIKPLLTSYNNLEYDLKAGRRGERYS-HLHGILKAILGCEEVLVVNNNAAAVFLILHTF

Aquaeo NFISEIANGYSNLEYNLEEGKRGSRIA-HIEKYLNELTGAESSFVVNNNAGAVFLVLNTL

Cloper EAVALAASSYSNLEYDLEKGERGSRYS-LIEGIIKDITGAEAALVVNNNAAAIMLVLNSL

Despsy ERLAQTASYYSNLEFDLATGKRGSRYS-LVEDTICELTGAEAALVVNNNAAAVFLALDAL

Mycavi -----AAGGATDVEFDLATGRRARRGRGALAALARAVPAAGGVHVVNNNAAALLLVALTL

Pseaer EAITLAARYPLNLEFDLASGKRGDRDD-LIAGLIRELTGAEAVTVVNNNAAAVLLALNSL

Pseput EAMQTAARYPLNLEFDLATGKRGDRDD-LIEGLIRELTGAEAVTVVNNNAAAVLLALNSL

Burpse RAVVQALTQPMNLEFDLATGRRGDRDD-LIGALACELTGAEAATVVNNNAAAVLLVLSAL

Burmal RAVVQALTQPMNLEFDLATGRRGDRDD-LIGALACELTGAEAATVVNNNAAAVLLVLSAL

Sinmel DAAVDAMREAAALEFDLDSGGRGERDS-HLRELLCELTGAEDATVVNNNAAAVLIALNSV

Haeinf QAALSAMQKNVSLEYDLDEGKRSHRDN-YISELLCKLTGAEAACIVNNNAAAVLLMLATF

Mansuc QAALTAMRQNVALEYDLEAGKRSHRDN-YVSELLHELTGAQAACVVNNNAAAVLLMLATF

Pasmul HAALTSMSHNVALEYDLEEGKRSHRDH-YISDLLCQLTGAEAACIVNNNAAAVLLMLATF

Haeduc TAASDAMRHHSALEFDIEAGKRSHRDN-TISQLLCEITGAEAACVVNNNAAAVLLMLATF

Yerpes AAVTDAMRSAVTLEYSLEGAGRGHRDR-AVADLLCALTGAEDACIVNNNAAAVFLLLTVM

Yerpse AAVTDAMRSAVTLEYSLEGAGRGHRDR-AVADLLCALTGAEDACIVNNNAAAVFLLLTVM

Saltyp EAVTQAMRAPVTLEYDLDGAGRGHRDR-ALATLLCRITGAEDACIVNNNAAAVLLMLAAT

Shifle EAVAQAMRSPVTLEYDLDDAGRGHRDR-ALAQLLCRITGAEDACIVNNNAAAVLLMLAAT

Esccol EAVAQAMRSPVTLEYDLDDAGRGHRDR-ALAQLLCRITGAEDACIVNNNAAAVLLMLAAT

Pholum EAVTQVMRSPVTLEYSLNNAERGHRDH-ALADLLCELTGAEDACIVNNNAAAVLLLLATV

Sheone RAVTSVMRYPTPLEFELAAGERGHRDN-AISGLIQRLTGAEACCVVNNNAAAVLLMLSAV

Desvul EAVAAACAHYSNLEFDLDTGERGSRYS-HVEQLLCRVTGAEAGLVVNNNAAAVLLVLDTL

Geosul QRVDDTAYGYSNLEFDLERGERGSRYS-HVEKLLCELTGAEAALVVNNNAAAVLLALSAL

Symthe AAIVDIAGRYNNLELDLSTGERGSRYA-HVEALLAELTGAEAAMVVNNNAAAVLLMLSAL

Theten EHLLDIAFCYSTLEYDVEKGERGSRYS-HVEKLLCELLDVEAALVVNNNAAAVLLALNTL

Treden DRAKETASGYSSIEMDLRDGNRGKRFE-FLNDCMSLLTGAEDALILNNNAAAVFLMLKAL

Phopro QAVTEVNTHYSNLEIDLTTGKRGKRKG-LLQQLMHYLVKGEDTLVVNNNASSIYLMLHEI

:* .: . *. * : ::****.:: :

Helhep AAHKEVIISRGELVEIGGSFRIPEVMKSASSILCEVGATNKTHLKDYENAINEQSAMIMK

Camjej CYDKEVISSRGELVEIGGSFRVPEVIKAAGVKLCEVGTSNKTHLKDYEQAINENTALILK

Wolsuc AQNQEAIISRGELIEIGGSFRIPEVMKNAGAILKEVGTTNKTHRRDYEEAITPQSALLMK

Aquaeo AEGKEVIISRGELVEIGGSFRIPDIMKKSGAILREVGTTNKTKVSDYEGAINQNTALLMK

Cloper CENKEVIVSRGELVEIGGSFRIPEVMNFSRAKLVEVGTTNRTHLYDYEDAITEETGAFLK

Despsy CRGTEVIVSRGQLVEIGGSFRIPDVMARSGADLVEVGATNRTHLYDYEDAISEQTSMLLR

Mycavi APGREIVLSRGELVEIGDGFRIPELLESTGARLREVGTTNRTGLRDYTEALGPQTGFVLK

Pseaer GARKEGIISRGELIEIGGAFRIPDIMARAGVRLHEVGTTNRTHAKDYEAAIGPRSGLLMR

Pseput GARKEGIISRGELIEIGGAFRIPDIMARAGVKLHEVGTTNRTHARDYEAAIGPRTGLLMR

Burpse APRREVVVSRGELVEIGGAFRIPDIMSRAGAKLREVGTTNRTHLADYADAIGPRTALLMK

Burmal APRREVVVSRGELVEIGGAFRIPDIMSRAGAKLREVGTTNRTHLADYADAIGPRTALLMK

Sinmel GAGRQAIVSRGELIEIGGAFRMPDIMERAGVDLVEVGTTNRTHAKDYVKAIGPETALILK

Haeinf AQGKEVIISRGELIEIGGAFRIPDIMEQAGCHLVEVGTTNRTHLKDYRNAITENTAFLMK

Mansuc AQGKEVIISRGELIEIGGAFRIPDIMAQAGCKLVEVGTTNRTHLNDYRRAINENTALLMK

Pasmul AQGKEVIISRGELIEIGGAFRIPDIMQQAGCKLVEVGTTNRTHLSDYRHAINENTAFLMK

Haeduc AKGKEVIVSRGELVEIGGAFRIPDIMQQAGCKLVEVGTTNRTHLKDYRAAINENTALLMK

Yerpes AAGKQVVVSRGELVEIGGAFRIPDVMRQAGCELVEVGTTNRTHLKDYRQAINENTGLLMK

Yerpse AAGKQVVVSRGELVEIGGAFRIPDVMRQAGCDLVEVGTTNRTHLKDYRQAINENTGLLMK

Saltyp ASGKEVVVSRGELVEIGGAFRIPDVMRQAGCTLHEVGTTNRTHAKDYRQAVNENTGLLMK

Shifle ASGKEVVVSRGELVEIGGAFRIPDVMRQAGCTLHEVGTTNRTHANDYRQAVNENTALLMK

Esccol ASGKEVVVSRGELVEIGGAFRIPDVMRQAGCTLHEVGTTNRTHANDYRQAVNENTALLMK

Pholum ASGKQVVVSRGELVEIGGAFRIPDVMVQAGCRLVEVGTTNRTHLKDYRQAINEETALLMK

Sheone AAGKEVIVSRGELVEIGGAFRIPDIMRQAGCTLVEVGSTNRTHLKDYEQAITENTAAIMK

Desvul CKGGEVVVSRGQLVEIGGSFRIPDVMEKSGATLREVGATNRTHLRDYENAINEKTVALLR

Geosul AGGKEVIVSRGELVEIGGSFRIPDVMQQGGAVLREVGTTNRTHSRDYRQAVTSETGLLLK

Symthe AAGKEVVVSRGELVEIGGSFRVPDVMKQGGARLVEVGTTNKTHPRDYEEAIGPETCALLK

Theten AKGKEVIVSRGQLIEIGGSFRIPDVMLQSGAILKEVGTTNKTYDFDYINAITENTALLLK

Treden AGGKEVIVSRGQQVQIGGGFRIPEILEMAGCKLIEVGTTNITSLKDIQKAINENTAMVLW

Phopro AKGKEVIVSRGEQIQIGGGFRIPDILALSGAKLVEVGTTNITTVDDYLDAVTDNTAMVLI

: : ***: ::**..**:*::: * ***::* * * *: .: .:

Helhep VHQSNFKQIGFVKQCHIKEIIHLAQKHHLIDYFDLGSGHIGALP---LA-DEPSVREICK

Camjej THKSNFALMGFHSEVNIKDLHELAKEKELLSYYDLGSGWCENLNEKLIK-NEPKIRKLVQ

Wolsuc VHKSNYDIVGFTQEVDLQELIELSQKHNLIDYYDLGSGFLESVP---FT-NEPTLKKIAS

Aquaeo VHKSNFYMEGFVEEVKLEDLVKLGHKYGIPTYYDAGSGLLINLKEFGISVDEPNFRDCIS

Cloper VHSSNFKIVGFTKSVSANDICNLAKEKGIPVIEDIGSGVLIDLSKYGLE-KEPTVIESLE

Despsy VHTSNFRIIGFTSEVPAEEMVGLARKKNLLTMEDLGSGSLIDLTSYGFP-KEPTVQELVK

Mycavi VHPSNFHVTGFTSAVGVAALAAELRTRDIPLVVDIGSGLLAP--HPVLP-DEPDATSTLA

Pseaer VHTSNYSVQGFTASVPTAQLAAIAHGHGLPLLEDLGSGTLVDLTRWGLP-KEPTVQEALA

Pseput VHCSNYSIQGFTTQVPTAELARIAHQHELPLLEDLGSGSLLDLTRWGLP-AEPTVRQALA

Burpse VHCSNYAISGFTKEVALRELAPLARERGVPVAVDLGSGTLVDLTRWGLP-KETTVRETVE

Burmal VHCSNYAISGFTKEVALRELAPLARERGVPVAVDLGSGTLVDLTRWGLP-KETTVRETVE

Sinmel VHTSNYRIEGFTAEVPGAELAAIAHERGVVLLNDLGSGSLVDLSRYGLG-REPTVREAVA

Haeinf VHSSNYQICGFTSSVSEEELTELGQEMNVPVVTDLGSGALVDLSQYGLP-KEPTVQEKIA

Mansuc VHSSNYQICGFTCEVSEQELVELGKEFNIPVVTDLGSGALTDLSRYDLP-KEPTVQEKLV

Pasmul VHSSNYQICGFTKSVTEAELVTLAREFNLPVMTDLGSGALVDLAQYGLP-KEPTVQEKWA

Haeduc VHTSNYQIQGFTHSVSEQQLVELGQEVGLPVMSDLGSGSLIDMATFGLP-AEPLVQQKVA

Yerpes VHTSNYSIEGFTAAVSEQQLAALGQECSIPTATDLGSGSLVDMTRYGLP-AEPMPQQLIA

Yerpse VHTSNYSIEGFTAAVSEQQLAALGQECSIPTATDLGSGSLVDMTRYGLP-AEPMPQQLIA

Saltyp VHTSNYSIEGFTKTVEEAELAEIGRELDIPVVADLGSGSLVDLSQYGLP-KEPMPQQLIA

Shifle VHTSNYSIQGFTKAIDEAELVALGKELDVPVVTDLGSGSLVDLSQYGLP-KEPMPQELIA

Esccol VHTSNYSIQGFTKAIDEAELVALGKELDVPVVTDLGSGSLVDLSQYGLP-KEPMPQELIA

Pholum VHTSNYNIDGFTAEVSGRELATLGIASQIPTAIDLGSGSMINMVQYGLP-AEPMPQDYLN

Sheone VHTSNYHISGFTAAVEEARLGQLCRERGILLISDLGSGSLTDLRRFGLK-QEPTPQAMLA

Desvul VHTSNYRVVGFHKEVPLDELVALGRARDLPVIEDLGSGSFLDFTPWGLP-GEPTVQSVVG

Geosul VHSSNFAVVGFTAEVTAPEMVAIGRELGLPVMADIGSGCLIDLSRFGIR-GEPTVQEFVS

Symthe VHRSNFRLEGFTAEVSVAELARIAHRHGLPCLVDWGSGVMLDLSRYGLP-HEATMPELIA

Theten VHTSNYRIVGFTHDIATEELVQIGRKYDIPTMEDLGSGVMVDLREYGLP-HEPTVQEVVK

Treden VHTSNYKIRGFTEQPSISEIKAILPQ-NIILAVDQGSGNL----SLNVP-EEPTVSSIIK

Phopro VHTSNFKIRGFTQSPDIKDLADLLPD-HVVLAVDQGSGTTTE----NLP-DEPSVMSYIK

.* **: ** : : * *** . *.

Helhep YKPSLLSFSGDKLLGGPQVGIIIGKSQLIAQLKHNQLLRALRVDKFSILALQATLKAYQN

Camjej E-CDILSFSGDKLFGSVQAGIILGKKELIEKLKQNQLLRMLRVDKLTLSFLNESLKAYLQ

Wolsuc LSPSLVSFSGDKLLGGAQAGIIFGKKSLIDRLKKNQLLRMLRVDKFTLAALEATLRAHLL

Aquaeo LGIDLVSGSGDKLLGGPQAGIIVGKKNLIEKIKKNPIARALRIDKLTLSGLEMTLKLYFE

Cloper SGVDIVTFSGDKMLGGAQAGIIVGKKKFIDKIKKNQLTRALRVDKFTLAALEITLKHYLN

Despsy AGVDVVTFSGDKLLGGPQAGIIVGSKSVVERIKKSPMNRAFRVDKFTLAALEVVLRSYYD

Mycavi DGADLVTASGDKLLGGPQAGLLFGRGDLVERLRRHPAARALRVDKLTLAALEATLTGPPP

Pseaer DGADIVTFSGDKLLGGPQAGLILGNRELIGRIKKNPLKRALRVDKLTLAALEAVLGLYRD

Pseput DGADIVTFSGDKLLGGPQAGIIVGHKDLITRIKKNPLKRALRVDKITLAALEAVLALYRN

Burpse AGADLVTFSGDKLLGGPQAGLIVGRRDLIAKIKKHPLKRALRVGKLTLAALEPVLRLYQT

Burmal AGADLVTFSGDKLLGGPQAGLIVGRRDLIAKIKKHPLKRALRVGKLTLAALEPVLRLYQT

Sinmel EGADLVTFSGDKLLGGPQAGFIVGRRDLIAEINRNPLKRALRVDKIRIAATAATLKLYRD

Haeinf QGVDLVSFSGDKLLGGVQAGIIVGKKEWIEQLQAHPLKRVLRCDKVILAGLEATLRLYLN

Mansuc QGADLISFSGDKLLGGPQAGIIVGKKELIQQLQSHPLKRVLRCDKVILAAMEATLRLYLQ

Pasmul QGVELISFSGDKLLGGPQAGIIVGKKAWIDRLQTHPLKRALRCDKVILAGLEATLRLYLQ

Haeduc SGVALVTFSADKLLGGPQAGIIVGKKADIEALQAHPLKRVLRCDKVILAGLEATLRHYLL

Yerpes AGVDLVTFSGDKLLGGPQAGIILGKKQWIERLQQHPLKRALRADKMTLAALDATLRLYQQ

Yerpse AGVDLVTFSGDKLLGGPQAGIILGKKQWIERLQQHPLKRALRADKMTLAALDATLRLYQQ

Saltyp AGVSLVSFSGDKLLGGPQAGIIVGKKAMIAQLQSHPLKRALRADKMTLAALEATLRLYLH

Shifle AGVSLVSFSGDKLLGGPQAGIIVGKKEMIARLQSHPLKRALRADKMTLAALEATLRLYLH

Esccol AGVSLVSFSGDKLLGGPQAGIIVGKKEMIARLQSHPLKRALRADKMTLAALEATLRLYLH

Pholum QGIDLVTFSGDKLLGGPQAGIILGKKHWIEAIQRHPLKRALRADKMTLAALEATLRLYQR

Sheone DGVDLVSFSGDKLLGGPQSGLIVGKQALINKLQSHPLKRALRCDKLILAALEATLIHYLN

Desvul AGPDVITFSGDKVLGGPQAGLIVGRRQWIDRIKRNPLNRALRIDKMTLAALEATLRLYLD

Geosul VGVDVVTFSGDKLLGGPQAGIIVGRKAYVDPLKKHPLLRAIRIDKLTLAALEGTLRLYRD

Symthe AGADLVTFSGDKLLGAPQGGFIVGRRDLVERCRRHPLTRAVRVDKLTLAGIEATLKLYLE

Theten AGVDIVTFSGDKLLGGPQAGIIVGKKKYIDLMKKNPLTRALRVDKLCLVSLECVLRIYRD

Treden EGADLVCFSGDKILGGPQAGWIVGKKNLVQTVAKNQLMRTYRVGRAVASLMQECLILYLN

Phopro AGADLVCFSGDKIIGGPQAGMVTGKQSLIKRLEKNPMMRAFRPSRIVYSLLEELAVRKLN

:: *.**::*. * * : * : * * .:

Helhep KEY--HKIPTLAMLAFTPKELESKAKNLKKRILASSIA-SELEVEVIPLHSLAGGGSVPH

Camjej KDY--EKIITLKLLNDDLSFIEKKALRVQKEL--------KFQTQLKKSKSLVGGGSMPD

Wolsuc HDY--EKIPTLKMAHLSLEELEERAKKLKSRV-------HGYESQILQTQGYAGGGALPN

Aquaeo --KRYEDIPVIRMLTQDEKALRQKAKRLEKLL-KDIPG---LKISVIKDKAKPGGGSLPE

Cloper EKEAIEKIPTLYMMTLDLNEIKERANRLYKNL-EG-LN-KFYKFSIEEGESTVGGGSMPD

Despsy SRQALQEIPTLRMLTTGKDVLLKRARRISRRL-SAKLK-EKCTLRIVPTMSRVGGGAFPE

Mycavi P--------VAAALDADVGRLRARAQSLAAAL-PA-----DADAVALDCVAAVGGGGAPG

Pseaer PDRLAERLTTLRLLSRPAAEIRAQAERLAPAL-GEALG-EGWEVAVVDALGMIGSGAQPV

Pseput PDRLAERLPSLRLLTRSQAEIQAQAERLAPEL-QARLG-EQWAISVEPALGMIGSGSQPV

Burpse PEFLRERLTTLRLLTRAQADIAATAERVRPAL-QRALG-AAFAVDVEPMFSQIGSGALPV

Burmal PEFLRERLTTLRLLTRAQADIAATAERVRPAL-QRALG-AAFAVDVEPMFSQIGSGALPV

Sinmel PDRLASRLPTLFMLSRVQAEVRAQAERLAPQV-GAMLAPSGYAVEVCSCSSQIGSGALPV

Haeinf PEKLTEKLPTLRLLTQPLKQLKINAMRLKERL-ESRLN-SQFELQIEASQAQIGSGSQPM

Mansuc PEKLTEKLTSLRLLTQPLEQLRQQAEQLKAKL-ENLLK-DDFLLQIESSLAQIGSGSQPM

Pasmul PEKLSQHLTTLRLLTQPVEALHEQAEQLQSVL-LTKLT-TDYSVAITNSVAQIGSGSQPM

Haeduc PDQLTTHLPTLSLLTQSIDHLRVKATRLQNSL-SKRLD-ARYHLQIEQSLAQIGSGALPT

Yerpes PDRLVEQLPSLRLLTRPASEIAACAQRLLAPL-IACYG-TDFTLDIESCWSQIGSGSLPV

Yerpse PDRLVEQLPSLRLLTRPASEIAACAQRLLAPL-IACYG-TDFTLDIESCWSQIGSGSLPV

Saltyp PEALAEKLPTLRLLTRSEASIREQAQRLQARL-AARYG-DEFALEVKPCLSQIGSGSLPV

Shifle PEALSEKLPTLRLLTRSAEVIQIQAQRLQAPL-VAHYG-AEFAVQVMPCLSQIGSGSLPV

Esccol PEALSEKLPTLRLLTRSAEVIQIQAQRLQAPL-AAHYG-AEFAVQVMPCLSQIGSGSLPV

Pholum PEQLCQQLPTLRLLTRSQQQMHDMAQRLLPQL-QAHYG-DQFIVRDEPCYSQIGSGSLPV

Sheone PETLDKELPIMAKFARSQAELRQIGERLQQAL-APLFT-PSYGLELVECQTQVGSGSQPD

Desvul PERARNEVPTLRMMTASPDELARRARRLAARL-RKALG-DAARVGTVPGVSRVGGGSFPE

Geosul ERQALAEVPTLRMLTASAEELRLRARQFMRRL-RRGVP-SSVRLASLDGVSQVGGGAYPL

Symthe PERAAERIPAVRMLTASREQIAPRAEALAGKL-RA----AGIACEVIDGVSRAGGGALPG

Theten SNPV-EAIPTLKMLTAKPSQLYEKAAILNKLV-LTIPK---VKSKVVEITSLSGGGSLPE

Treden GGESAAQ----RALLLDQKKIKKRAEKIIASL-KSGAG------ELVQKNFSLGGGSTPD

Phopro QLKTGQGIAE-RSITQAEVTLENKAQQLAEGF--------GERLRIVRSEMTVGGGSLPD

: . . . *.*. *

Helhep LSFDSFGISLQAKH------LQVQDFEFALRA--LGLISCIQGEKILLDVRTLLEGDEER

Camjej KSLDTYILTFQGD---------ALKLQTRFRK--ENIIGRIENDEFVLDFRTIRENELQK

Wolsuc QSFFSIALALCHPQK------SPMELEQSLRA--RGVIARIEQERVLLDMRTIFTSQLES

Aquaeo LELPTYCVAIRHDR------LSSQELSRRLRLAEPPIVCRIREDQLLFDMRTVFHEDLKT

Cloper SKLSTYLLRIDSDRI------NEVNLERELREYKIPIITRVYKGAVYIDLRTILEDDYEV

Despsy HDLASWAVAFRPEHI------RLSEIEKRLRKLNTPIIGRLENEKFLLDVRTIQDDEVGL

Mycavi VELPSAAVSLP------------ERYAAALRAGRPPVVGRLEGGRCLLDLRTVSPEDDEL

Pseaer ARLASAALCLRPRQPRRLRGRALRNLEEALRGLPLPVIGRLDDDALWLDLRQLD--DEPA

Pseput ARLPSAALCLRPQVSKKLRGRSLHVLERALRDLPVPVLGRIDDDALWLDLRQLD--DEAQ

Burpse DQLPSYGLVVRASGGKR-RGRALAQLDAHLRGWPRPVLGRIADDALRLDLRCLEAGDEAA

Burmal DQLPSYGLVVRASGGKR-RGRALAQLDAHLRGWPRPVLGRIADDALRLDLRCLEAGDEAA

Sinmel DTIPSAGLRIVGSS-----GSALEALAALFRSLSRPILGRLRDGALVLDLRCLS--DEAE

Haeinf ERIPSVAVTIAEKT-----NAKLSALSARFKQLSQPIIGRMENGKIWLDLRSLA--DIET

Mansuc AKIPSIAVTIAEKN-----SEKLTALLARFKKLSTPIIARVENDKIRLDLRSVT--AIET

Pasmul ATIPSVAVTISTEK-----AGKLTALLQRFKALPQPIICRVEKEKIWLDLRGLA--DIDS

Haeduc ERLASLAVTITAPT-----QRDLLQLEQQFKMLKYPIIGRFAEQKLWLDLRSVA--QFDQ

Yerpes DRLPSWALTFTPKDG---RGSTLEALTARWRTLTKPVIGRVADGRLWLDLRCLE--DEAA

Yerpse DRLPSWALTFTPKDG---RGSTLEALTARWRTLTKPVIGRVADGRLWLDLRCLE--DEAA

Saltyp DRLPSAAMTFTPHDG---RGSRLEALAARWRTLPVPVIGRIYDGRLWLDMRCLE--DESR

Shifle DRLPSAALTFTPHDG---RGSHLESLAARWRELPVPVIGRIYDGRLWLDLRCLE--DEQR

Esccol DRLPSAALTFTPHDG---RGSHLESLAARWRELPVPVIGRIYDGRLWLDLRCLE--DEQR

Pholum DRLPSWALTFAAVEG---QGSSLERLARCWRGLAKPVLGRISGGRLWLDLRCLE--DEKA

Sheone TFLPSIGLCFNAQE-----GGSLTLLEQHFKQAQRPVIGRMTQDQLRLDLRGID--DEAE

Desvul RDLPTTLVEVAPA------SCTATVLKTRLLDTDPPLVGRLENDTFRLDPRTLDDVEFAP

Geosul LELPTTLIAVDVD------GVSPQEMEARLRRMTVPVVGRINRGRFLLDARTLLDDDAPA

Symthe VDLPTRLVAVAHP--------APQEVEARLRAFDPPVMVRIQAGLLLLDPRTLWDDELDV

Theten ESLPSYGITLEVE------GFDTEDLERRLRIRDIPIITRIVDGVVTIDVRTLLEGDEEV

Treden TGFSSWAVKLNTK-------KPCEKLKTNLREMPIPIIGFIEEDSFYIHPASIEEKYDEY

Phopro QQSPSSSVEVNAISL------SADKLLEIMRNWPLPVIGVIKHDRVQLNLATISDDEITY

: : . :: . :. :

Helhep IMEILGEVLSS-------------------

Camjej LILTINQMENL-------------------

Wolsuc LAQILNEVF---------------------

Aquaeo IKKTLQELLSI-------------------

Cloper IFNALKEIGEK-------------------

Despsy LCSLLLEFFLEAS-----------------

Mycavi LAAAVRACSS--------------------

Pseaer FLAQLPRLRSELS-----------------

Pseput WLAQLPALQLGPVQ----------------

Burpse FIAQCAQAPTGPRA----------------

Burmal FIAQCAQAPTGPRA----------------

Sinmel FLKTLSEGSGDAVA----------------

Haeinf LLNTLDEL----------------------

Mansuc LLITLEELNQDQ------------------

Pasmul LLKTIMQL----------------------

Haeduc LIEMLEEK----------------------

Yerpes LLRELAS-----------------------

Yerpse LLRELAS-----------------------

Saltyp FMEMMLK-----------------------

Shifle FLEMLLK-----------------------

Esccol FLEMLLK-----------------------

Pholum LLQALLL-----------------------

Sheone LLAELSTLGVQL------------------

Desvul VATALRQALGL-------------------

Geosul VISALRELAS--------------------

Symthe VVSALRSAIV--------------------

Theten ILHALEEITGVCQ-----------------

Treden VIEALNKCL---------------------

Phopro LRQQLAALFDLQLRGSCSNVTDTKKVEAAI

**SELB**

CLUSTAL FORMAT for T-COFFEE Version_1.37, CPU=0.00 sec, SCORE=41, Nseq=35, Len=828

Pseaer -------------VIVGTAGHIDHGKTSLLRALTGIEG----DRRPA----ERQRGITID

Pseput -------------VIVGTAGHIDHGKTALLLALTGQAG----DQRQE----ERARGMTID

Burmal -------------MIVGTAGHIDHGKTTLVRALTGVDT----DRLKE----EKARGISIE

Burpse -------------MIVGTAGHIDHGKTTLVRALTGVDT----DRLKE----EKARGISIE

Haeinf -------------MIIVTSGHVDHGKTALLKALTGTST----AHLPE----EKKRGMTID

Pasmul -------------MIIVTSGHVDHGKTALLQALTGTHT----AHLPE----EKKRGMTID

Mansuc -------------MIIVTSGHVDHGKTALLQALTGMNT----AHLPE----EKKRGMTID

Haeduc -------------MIFVTAGHVNHGKTSLLQALTGMNT----AHLPE----EKKRGLTID

Pholum -------------MIFATAGHVDHGKTTLIQAITGVNT----AHLPE----EKKRGMTID

Yerpes -------------MIIVTAGHVDHGKTTLLQAITGVNA----DRLPE----EKQRGMTID

Yerpse -------------MIIVTAGHVDHGKTTLLQAITGVNA----DRLPE----EKQRGMTID

Saltyp -------------MIIATAGHVDHGKTTLLQAITGINA----DRLPE----EKKRGMTID

Shifle -------------MIIATAGHVDHGKTTLLQAITGVNA----DRLPE----EKKRGMTID

Esccol -------------MIIATAGHVDHGKTTLLQAITGVNA----DRLPE----EKKRGMTID

Sheone -------------MIMVTAGHVDHGKSTLIRALTGMNT----DRLPE----EKRRGMTID

Mycavi ------------VFVLATAGHVDHGKSTLLHRLTGMWP----DRLAE----EQRRGLTID

Treden -----------MPYILGTAGHVDHGKTALVKRLTGIET----SHLPE----EKKRGMTIE

Phopro --------------------------------------------------------MTID

Metjan ----LVISMEMKNVNVGLFGHIDHGKTQLAKQLTEIASTSALDKPKE----SQKRGITID

Metmar --------MDFKNINLGIFGHIDHGKTTLSKVLTEIASTSAHDKLPE----SQKRGITID

Aquaeo ----------MKYILFATAGHVDHGKTTLIKTLTGIDT----DRLPE----EKKRGLSID

Helhep ------MSEGHNDIIVGLAGHIDHGKTTLIKALNGFDG----DSLEE----EKQRGITLD

Camjej ----------MKSVIIGTAGHIDHGKTSLIKALNGFEG----DSLKE----EQERQITIN

Wolsuc ----------MESYIVGTAGHIDHGKTALIRALNGFEG----DTTPE----EKKRGITVD

Sinmel -------------MIVGTAGHIDHGKTTLVKALTGVDT----DRLKE----EKARGITID

Metkan ----------VPVVHVGLFGHIDHGKTALAAQLTEKPSTAALDKHPE----EKERGITID

Cloper ----------MKHVIIGTSGHIDHGKTTLIKALTGRET----DKIDE----EKKRGISIN

Theten ----------VKNIIIGTAGHIDHGKTTLIKALTGKDT----DRLKE----EKLRGITTD

Desvul -----------MPVIMGTAGHIDHGKTSLVRALTGIDC----DRLDE----EKRRGITIE

Geosul ----------MKHLILGTAGHIDHGKTSLVRALTGIDT----DRLPE----EKARGITIE

Despsy ----------MREIVLGTAGHVDHGKTSFVRALTGIET----DRLKE----EKKRGITIE

Symthe ----------MKHYIIGTAGHVDHGKSALIRALTGVET----DRLPE----EKERGISID

Homsap ------MAGRRVNVNVGVLGHIDSGKTALARALSTTASTAAFDKQPQ----SRERGITLD

Dromel ---------MPINFNIGLLGHVDSGKTTLAKALSSISSTAAFDKNPQ----SVERGITLD

Caeele MTSPPSTSSSVGPLNLGILGHVDSGKTTLTRRIAELGSTSAFDAHATSTTTDGIRRNTLD

: :

Pseaer LGYLYADLGDG------------------------SP-TGFIDVPGHERFVHNMLAGASG

Pseput LGYRYAALSEG------------------------APLTGFIDVPGHERFIHNMLAGAHG

Burmal LGYAYTPLPNG------------------------DV-LGFIDVPGHEKLVHTMAAGACG

Burpse LGYAYTPLPNG------------------------DV-LGFIDVPGHEKLVHTMAAGACG

Haeinf LGYAYLPLE-------------------------NKV-LGFIDVPGHEKFLSNMLAGLGG

Pasmul LGYAYLPLEKN--------------------GKIDRT-LGFIDVPGHEKFLVNMLAGLGG

Mansuc LGYAYLPVG-------------------------DKI-LGFIDVPGHEKFLANMLAGLGG

Haeduc LGYAYLAIEDE--------------------MAKVER-LGFIDVPGHQKFMSNMLAGLGG

Pholum LGYAYWPQPDG------------------------SS-VGFIDVPGHEKFLANMLAGIGG

Yerpes LGYAYWPLPDG------------------------RI-MGFIDVPGHEKFLANMLAGGGG

Yerpse LGYAYWPLPDG------------------------RI-MGFIDVPGHEKFLANMLAGVGG

Saltyp LGYAYWPQPDG------------------------RV-LGFIDVPGHEKFLSNMLAGVGG

Shifle LGYAYWPQPDG------------------------RV-PGFIDVPGHEKFLSNMLAGVGG

Esccol LGYAYWPQPDG------------------------RV-PGFIDVPGHEKFLSNMLAGVGG

Sheone LGYAFMPLRDG------------------------TR-LAFIDVPGHEKFINNMLVGVSH

Mycavi LGFVWTELA-G------------------------RR-LAFVDVPGHERFVANMLAGVGP

Treden LGFASLEDPVH------------------------GT-VGIVDVPGHERFIRNMVAGTWG

Phopro LGFAFFKHNNG------------------------EA-VGVIDVPGHERFIRNMVAGVWS

Metjan LGFSSFTLDR-------------------------YR-ITLVDAPGHSELIRTAIGAGNI

Metmar IGFSAFKLEN-------------------------YR-ITLVDAPGHADLIRAVVSAADI

Aquaeo IGFAYIDFPD-----------------------INTR-LEIIDVPGHERFIKNAIAGICS

Helhep LSFSHLHLPS-------------------------RN-VAFIDVPGHNKLVKNMIAGAFG

Camjej LSFSNLKLKD-------------------------KN-ISFIDVPGHKDLVKTMVSGAFG

Wolsuc LSFSNLFLRE-------------------------KN-IAFIDVPGHEKLVKNMIAGAFG

Sinmel LGFAYARFAKD------------------------AV-TGFVDVPGHERFIHTMLAGAGG

Metkan LGFSSFELGD-------------------------YT-VTLVDAPGHADLIRTVVAGAEI

Cloper LGFTFFDLPSG------------------------KR-AGIIDVPGHEKFIKNMLAGATS

Theten LGFAYFDLPSG------------------------IR-AGIIDVPGHEKFIKNMLAGAHG

Desvul LGFAFCDLPGG------------------------GR-LGVVDVPGHEKFVRNMVAGASG

Geosul LGFAHLELPGG------------------------LQ-FGIVDVPGHERFVRTMVAGVGG

Despsy LGFAYMDLPCG------------------------HR-LGIVDVPGHEKFVKNMVAGVSG

Symthe IGFARFPLPSG------------------------RR-AAVIDVPGHEKFVRNMLAGITG

Homsap LGFSCFSVPLPARLRSSLPEFQAAPEAEPEPGEPLLQ-VTLVDCPGHASLIRTIIGGAQI

Dromel LGFSGLLVDAPAHL----------------PQGEQLQ-FTFVDCPGHASLIRTIIGGAQI

Caeele LGFSTMTSLSG------------------------RR-LALIDCPGHSGLIRAVLAASTV

:.: .:* *** :: .

Pseaer IDCVLLVVAADDGLMPQTREHLAIVELLGIRRALVALTKIDRVEPQR----VQQ--VRTQ

Pseput IDLVLLVVAADDGVMPQTREHLAIIELLGIPQALVAISKCDRVEPSR----LAE--VQMQ

Burmal IDFALVVIAADDGVMPQTREHLSILQLLGVAHGALALTKCDRVDAAR----VAR--VRDE

Burpse IDFALVVIAADDGVMPQTREHLSILQLLGVAHGALALTKCDRVDAAR----VAR--VRDE

Haeinf VHYAMLIVAADEGVAVQTKEHLAILRQLQFHEIIVVITKADRTNSAQ----IES--LIQT

Pasmul IHYAMLIVAADEGVQAQTLEHLAILRLLQLEHILIVITKADRATLPQ----IEQ--LSQQ

Mansuc IHYAMLIVAADEGIQAQTKEHLAILRLLQIEKIMVVISKADRASSAK----IDE--LKTK

Haeduc IQHALLVVSAEEGIKPQTEEHIEILRLLKFEQIAVIVTKSDRANMSQ----MLA--LIAQ

Pholum IDHVLLVVACDDGVMAQTREHLAILRLIGRPAMTVALTKADRVETQR----IVE--VRQQ

Yerpes IDHALLVVACDDGVMAQTREHLAILRLSGRPALTVALTKADRVDDER----IAQ--VHQQ

Yerpse IDHALLVVACDDGVMAQTREHLAILRLSGRPALTVALTKADRVDDER----IAQ--VHQQ

Saltyp IDHALLVVACDDGVMAQTREHLQILQLTGNLQLTVALTKADRVDEAR----ISE--VREE

Shifle IDHALLVVACDDGVMAQTREHLAILQLTGNPMLTVALTKADRVDEAR----VDE--VERQ

Esccol IDHALLVVACDDGVMAQTREHLAILQLTGNPMLTVALTKADRVDEAR----VDE--VERQ

Sheone VRHALLVLACDDGVMPQTREHLQILALLPLNSLTLVLTKRDLVDDQT----AAK--LASE

Mycavi VPAVVFVVAATEGWMPQSEEHLAALDALRVRHGLLVISKADLADPGP----AVA-----Q

Treden LDAALLIVAADDGWMQMSSDHLRVLKAMKIESILLVITKSDLAEKDM----LEL--LIED

Phopro LDMVLFVVAADEGWMPMSTDHLKVITAMGIDNVTLVITKSDLVDKDM----LAL--VEEE

Metjan IDAALLVVDAKEGPKTQTGEHLLVLDLLNIPT-IVVINKIDIANDEE----IKR--TEMF

Metmar IDLALIVVDAKEGPKTQTGEHMLILDHFNIPI-IVVITKSDNAGTEE----IKR--TEMI

Aquaeo ASGLILVVDPNEGIMPQTIEHLRVAKSFGIKHGIAVLTKMDKVDEEL----AHI--AEEE

Helhep IDVLLLVIAANEGIMPQSIEHLLIADMLGISSCICVITKIDKLENPS----LELPRLESD

Camjej FSVCLFVVDINEGLKEQSLEHLEILKILDIKNIILVLSKCDLCEN------IEQK--SVE

Wolsuc FDAMLLVVSAVEGIKPQTLEHLHIAHLLEIPTLIVALTKSDLVDQET----LLK--RESE

Sinmel IDYAMLVVAADDGIKPQTLEHLAILDLLGVSRGLVAITKADLADPAR----LEN--LTDE

Metkan IDAAILVVAADEGPQVQTGEHLVVLNHLGIDRGVIALNKVDLVDEKT----VER--RIEE

Cloper LDVVLLIIALDEGIMPQTKEHLEILELLEVKKCIVALTKRDLVDEEW----AEM--IKED

Theten IDIVMLVIAADEGVMPQTKEHVDILSFLDIKAGIIVLTKCDLVEKDW----LLI--VEED

Desvul VDFVMLVIAADEGVMPQTREHLEICSLLGIRHGLVALTKVDMVDADW----LEL--AQDD

Geosul MDLVMLVIAADEGVMPQTREHLEICQLLGVKKGLVALTKSDMVDPDW----LEL--VVEE

Despsy MDILAFIIAADEGIMPQTKEHFEICQLMGVKQGLIILTKKDLVDPDW----LEM--VEEE

Symthe IDLVILVVAADEGVMPQTREHLDILRLLEISKGLVAITKIDLVDEEM----VEL--VEAD

Homsap IDLMMLVIDVTKGMQTQSAECLVIGQI-ACQKLVVVLNKIDLLPEGKRQAAIDK--MTKK

Dromel IDLMLLVVDAQKGKQTQTAECLIIGELLQ-KKLIVVINKIDVYPENQRASKLEK--LRLR

Caeele FDMAIVIIDVVAGIQPQTAEHLLLASKFCPNRVIIVLNKCDLAEKSK----ISE--SAKK

.:: * : : . :.* *

Pseaer VEHLLASG-PLA--RSPIFPLSSS--TG----------EGVEALREALGGLAGEVRE---

Pseput VSELLAPG-PYA--GARQFPLSSI--SG----------EGVDALRQALLEAEAGTRQ---

Burmal IRAWLAAS-PLA--DAPVFETRASEPDD----------AGVAALNAHLRDTALAWRA---

Burpse IRAWLAAS-PLA--DAPVFETRASEPDD----------AGVAALNAHLRDTALAWRA---

Haeinf IKQDY--S-FLR--NANYFVTSA--ETG----------QGISELRHYLANLAELA-----

Pasmul LKA--NHP-ILA--NSPVFITSA--KTE----------QGIDELRNYLAKLPNLA-----

Mansuc ILTDY--P-FLA--ESPFFVTSA--VNG----------RGIAELREFLTALPNPA-----

Haeduc LRTRY---PFLH--TSPCFYTST--VTG----------AGIDELKQYLINCATQNS----

Pholum IEHELATQ-GWS--ESPLFVTAA--ADK----------ESIVPLRQHLLQLHQQNKQ---

Yerpes ILQELVAQ-GWSAEQISLFVTAA--VTE----------RGIGELREHLAQCHQQRDP---

Yerpse ILQELVAQ-GWSAEQISLFVTAA--VTE----------RGIGELREHLAQCHQQRDP---

Saltyp VLAALDNY-GFA--DTVLFVTAA--NEG----------RGIAELRAHLQQLPARLHA---

Shifle VKEVLREY-GFA--EAKLFITAA--TEG----------RGIDALREHLLQLPEREHA---

Esccol VKEVLREY-GFA--EAKLFITAA--TEG----------RGMDALREHLLQLPEREHA---

Sheone GITLLAEY-GIE--ASGVFEVCASDAGD----------AGVETLKQHILDIAEQQST---

Mycavi ATERFAAT-SLA--GVPV-------AIG----------TDLERVRAELVALTDRLPP---

Treden ANAQCEKI-IGR--KLPAVAVSS--LTG----------SGIEELKAEITKLLSSSKK---

Phopro ALEQFLEIGGF---IPDSLSVSA--HQS----------LGIEALKHHICQKLDTLSRPVL

Metjan MKQILNSTINLK--NSKIIKISA--KTG----------EGIGELKKELKNLLDSLDI---

Metmar MKSILQSTQNLK--NSSIIPISA--KTG----------FGVEELKNLIINTLNNAEI---

Aquaeo LIAFLEKE-EM---NMEIVKVSA--VTG----------QGIEDLKNSIKKLLESINN---

Helhep IVALFKTC-DMKLYDTIALSLHSSTMPL------TNAYDSLSQLKR-LKTSLDSIPK---

Camjej ILEELKNL-DYP--ILKVFHTSI--KNN----------QGIEELKNYLYTIENKE-----

Wolsuc ILNTLKTLPSLH--LHRLLSVSI--YDP----------DSLERLKESLYELSKPA-----

Sinmel IGAVLSST-SLR--DAEILPVSV--AAG----------QGIELLKERLAAAECATAA---

Metkan IKRVLQGT-TLE--DAPIIPVSA--KIG----------EGIEDLKDALLEVLEPP-----

Cloper IKNYLKST-SFK--DATMIEVSS--KTK----------EGLNELITEIDSAVEEIEQ---

Theten VRENLKGT-FLE--NAPIVHVSS--VTG----------EGLDILVNTLDELAQKVKE---

Desvul VAGFLAGT-FLE--GAPIFPVSA--VTG----------QGLDTLREHLATLERELRP---

Geosul VRDYLAGS-FLE--EAPIVPVSS--RTG----------AGIEAVKAELARLAGQVDE---

Despsy IREFCQGS-FLE--DAPILPVSS--ASG----------EGLDEVRDVLDELVKRQSF---

Symthe VAEAVAGT-FLE--GAPVCRVSS--VTG----------EGLDHLLRTVDALLEETEP---

Homsap MQKTLENT-KFR--GAPIIPVAA--KPGGPEAPETEAPQGIPELIELLTSQISIP-----

Dromel LAKTLEAT-TFG-GQVPICAVSA--LQG----------THIAELREVLREAYFQP-----

Caeele VRKGLKSM-GVD-ENSPIVEMSL--ADG----------YFKEEMLQNLKLAIESRIF---

: :

Pseaer ------------------RSREGYFRLAVDRAFSVAGSGIVVTGTAFSGQVAVGDELLLG

Pseput ------------------RSVVGGFRLAVDRAFAVTGAGVVVTGTALAGRVSAGDTLLLG

Burmal ------------------RRDDGLFRLAVDRVFTLAGQGTVVTGTAVAGRVRTGDSLAVA

Burpse ------------------RRDDGLFRLAVDRVFTLAGQGTVVTGTAVAGRVRTGDSLAVA

Haeinf -------------------DTQKPFRYAIDRVFSVKGAGTVVTGTAFSGTVKVNDEIYLS

Pasmul -------------------DSNKPFRYAIDRVFSVKGVGTVVTGTAFSGRVQIDDELLLS

Mansuc -------------------DKDKPFRYAIDRIFTVKGAGTVVTGTAFSGKVKIDDELYLS

Haeduc -------------------QTAKPFRYAIDRIFNIKGAGLVVTGTAIAGQVKVGDELYLS

Pholum -----------------HKKLHQRFRLAIDRAFSVKGAGLVVTGTALAGRVRVGDTLWLT

Yerpes -----------------KGRLQRRFRLAIDRVFSVKGAGLVVTGTALAGQVAVGDTLWLT

Yerpse -----------------KGRLQRRFRLAIDRVFSVKGAGLVVTGTALAGQVAVGDTLWLT

Saltyp --------------------AQHRFRLAIDRAFTVKGAGLVVTGTALSGEVNVGDTLWLT

Shifle --------------------SQHSFRLAIDRAFTVKGAGLVVTGTALSGEVKVGDSLWLT

Esccol --------------------SQHSFRLAIDRAFTVKGAGLVVTGTALSGEVKVGDSLWLT

Sheone -----------------QAEDASSFRMTLDRAFSVKGVGCVVTGTVISGSVCVGDSLYSS

Mycavi ------------------PDPHADVRLWVDRSFTVRGAGTVVTGTLAAGTVRVGDELE--

Treden ------------------QNPPTP-FLYVDRVFVLKGIGTTVTGTLRGKGLHTGDSLQIY

Phopro DSSATALQDRHQKASQQKISQQQEGRLYIDRVFTVNGVGTTVTGSLCGGEFHVGQKLVMQ

Metjan -----------------KRDINSYLKMPIDHAFKIKGVGTVVTGTIHKGKVEVGDNLRIL

Metmar -----------------IRNTESYFKMPLDHAFPIKGAGTVVTGTINKGIVKVGDELKVL

Aquaeo ------------------LNKHKPLRIFVDSAFVVKGYGTVLRGSCFEGEVKEGDKVVVE

Helhep ----------------PQKIDFGLFLYYIDRSFSIKGAGCVVTGSVLSGECCVGDKLYAY

Camjej ------------------NDEELIFHYYIDRVFSLKGIGTVVTGSLNEGSITLNEKIICL

Wolsuc ------------------TRDLGFFRYYIDRSFSIKGAGCVVSGTVLSGKVEQNQKIWCC

Sinmel ------------------SAVGGRFRLAVDRSFTLSGAGTVVTGTVLSGSVGVGDQVTVS

Metkan -----------------NRDLDSPFRMPIDHAFHVKGAGTVVTGTVLTGRVEVGDELTLY

Cloper ------------------KDKEGHFRLAVDRSFSVSGFGTVATGTILSGSVKLGDLVQIN

Theten ------------------RSSEGIFRLPVDRVFSIAGFGTVVTGTLISGKIKVGDKVMIY

Desvul ------------------ERRTDLFRLPVDRVFTMRGHGTVVTGTMISGSLKVGDDVVLY

Geosul ------------------KKTEGPFRLPVDRVFTVTGFGTVVTGTLLSGAISVGDEVELL

Despsy ------------------NEIFGSFRLAVDRVFAMKGFGAVVTGTSLSGRAALGDELRFY

Symthe ------------------KDTTAFARLPIDRAFVRPGFGTVVTGTLVGGVIRQGDRMELL

Homsap -----------------TRDPSGPFLMSVDHCFSIKGQGTVMTGTILSGSISLGDSVEIP

Dromel -----------------QRNLADPLFMYVDHCFGIKGQGTVCTGTLLQGKVQVNNVIELP

Caeele ---------------EPKRDEEGEFLIAIDHCFAIKGQGTVLTGTVIRGVLRLNTEIEFP

:* * * * . *: . :

Pseaer NAGRPVRVRGLHAQNRPAQQAWAGQRVALNIAGERLALEQIQRGDWLL-QAALH-APTTR

Pseput KAGKVVRVRGLHAQNQAALVAEAGQRVALNIAAERLAVEQVHRGDWLV-PEWLH-APSVR

Burmal RTGETVRVRSIHAQNRATDVGHAGERCALNLAG--IDKAALARGDAIV-DARLA-TLSPR

Burpse RTGETVRVRSIHAQNRATDVGHAGERCALNLAG--IDKAALARGDAIV-DARLA-TLSPR

Haeinf T-GQKIRIKAIHAQNTSSEQGIAGQRLALNLNAD-LDRTPMKRGDWLL-QNEPL-PPTDR

Pasmul N-GQTVRIKNIHSQNQQTNVGNAGERLALNLNVD-LDRITIERGDWLL-SQQPL-PPTSR

Mansuc N-GGKVRVKNIHAQNRQNTEGLAGQRLALNINAD-LDRTQIERGDWFF-SQAPF-EPTER

Haeduc N-GESVRVKAIHAQNCIAERGSAGQRLALNLA-N-VEKEQLQRGNWLS-ALDPK-FATDR

Pholum GRDQQIRVRGLHAQNQQADTAQAGQRIALNIAGD-VSKEQVSRGDWLL-SRQPF-EHVEK

Yerpes GGDCPVRVRGIHAQNQPAQQAQAGQRIALNISGD-ISKQQINRGDWLL-AQPPL-PPVDR

Yerpse GGDCPVRVRGIHAQNQPAQQAQAGQRIALNISGD-ISKQQINRGDWLL-AQPPL-PPVDR

Saltyp GVNTPMRVRSLHAQNQPTDHAYAGQRIALNIAGD-AEKEQLNRGDWLL-SDAPAGEAFSR

Shifle GVNKPMRVRALHAQNQPTETANAGQRIALDIAGD-AEKEQINRGDWLL-ADVPP-EPFTR

Esccol GVNKPMRVRALHAQNQPTETANAGQRIALNIAGD-AEKEQINRGDWLL-ADVPP-EPFTR

Sheone GQKAKLRVRGIHCQGMEVHRALSGTRVALNLTGVD-NHRAPARGDWLS-SLPQA-DLCAR

Mycavi HAGRRVTVRGLQSLGRNEIQVGAVARVALNLRG--VDRRDIGRGDTVR-TPGAW-RDTAE

Treden PSNEECRIKSIQNHHKDVEKIEPGTRTALNLKL--GEKTNLERGMLLA-EKDSNFVLSGK

Phopro PSGTEVQVKSLQSYHQNIQTASPVSRVAIGLKG--IKKKDVQRGFCLL-ESKEGAFVANE

Metjan PINHEVKVKSIQCFKQDVSIAYAGDRVGMALMG--VEPESLFRGCILT-SEDTKLKVVDK

Metmar PINMSTKVRSIQCFKESVMEAKAGDRVGMAIQG--VESKQIYRGCILT-SKDTKLQTVDK

Aquaeo PIGVISRVRKMQNHGVFVKKAVAGERIALNLPE--VDAKKVKRGFLIL-KPESY-EKSNV

Helhep DIKQEVAVRGIQIHDKNATKATPSHRVALNLSG--ISHHQLKRGFLLS-QKGYL-RGFDS

Camjej DTQKELIVKNIQNHDTNLEQIKACNRVALSLN---CDYKELKKGYLLS-KKGYF-KAFKE

Wolsuc ELQKPLAIRGIEVHGSYTQSALPSQRAALNLTG--VSHTELERGFLLT-QKGYL-RGFDR

Sinmel PAGRSARVRSIHAQNQRAERGFAGQRCALNLAGEGISKDAITRGDMVV-DPHLH-APSDR

Metkan PIGKTVEVKSIQSFGKDKQEACAGDRVGIALRG--IREEEIERGFQLA-EEGSL-RVTRY

Cloper PSGIEARVRNIQVHDENVEIGEAGQRCALNLSG--VTKEEVTRGMVVC-TANTI-EPSYM

Theten PKMIESRVRNLQVHERNVECAFAGQRTAINLAN--VKVEEIERGDVIA-PPEAI-IPSTM

Desvul PEGRTSKVRGLQSHGGPVDKALAGRRTAVNLQG--VDVAEVQRGEVLA-LPGTL-FPAQR

Geosul PSGLSARVRGVQTHGRRGDAASAGQRVAVNLQG--VEHTEVGRGDIVV-PRGVY-RTTRA

Despsy PGELVAKVRGLQVHSEAVDYVEAGHRTAINLQG--VDIADIARGMVLA-HDGSL-QATYM

Symthe PLGIEVRVRGLQVHGEPVEQAQAGQRVAVNLAG--IERSDVRRGHVLC-APGAL-RPTRS

Homsap ALKVVKKVKSMQMFHMPITSAMQGDRLGICVT-Q-FDPKLLERGLVCA-PES--LHTVHA

Dromel ALGEQRKVKSIQMFRKNVTSASMGDRIGLCVT-Q-FNAKLLERG--IITQPGY-LKPIYA

Caeele ALNEKRRVKTLETWKQRVGQVAAGARAAFLVAPS-FDEHRFSR--CICGPLGT-LKSTKF

:: :. * .. : :

Pseaer VDIDFRLL---PDELRDFTHWTPVHLHLGTQDVTGRLALLEG----EQLEPG--------

Pseput VDIELYLL---PDEKRAFEHFSAVHVHLGTQDVTARVALLEG----ESLAAG--------

Burmal IDVELTLT---ADADLTISHWTPLHVHLGTLHRVAHVALLEG----ETLGPG--------

Burpse IDVELTLT---ADADLTITHWTPLHVHLGTLHRVAHVALLEG----ETLGPG--------

Haeinf ISVQI-------LAEVPLNESQPVHIYHGASRTTGKLTLLQG----KNAAKN--------

Pasmul ITVLL-------NAETTLQESQPIHLYHASARTTGKLNLLQH----KTLLPA--------

Mansuc FTIQL-------TAETSLTENQPVHVYHAASRTTGKLALLIE----KAIYPG--------

Haeduc ISIQL-------TASTDLRENQVVHLYHFASHTMGKLNLLDA----KQAVRN--------

Pholum VLVEV-------ETDEPLRNWQSLHIHHAASHITGRISLLNP----LSDK----------

Yerpes VLVTV-------DADTPLQHWQPLHLYHGARHITGRFSLLGH----QQLIDDQ-------

Yerpse VLVTV-------DADTPLQHWQPLHLYHGARHITGRFSLLGH----QQLIDDQQPLIDDQ

Saltyp VIVSL-------ALHAPLSQWQPLHIHHAASHVTGRVSLLE-------------------

Shifle VIVEL-------QTHAPLTQWQPLHIHHAASHVTGRVSLLED------------------

Esccol VIVEL-------QTHTPLTQWQPLHIHHAASHVTGRVSLLED------------------

Sheone PVVHI-------RSFEPLTHWQNVHCHHGADHTLGRVSLLDE-------ADGQ-------

Mycavi IDVALRGP---GTAEDTL-HSQLV-LHVGSAAVPVRVRRLG-------------------

Treden -ELLVRIDEVFTNKEGGFKNHAEIEIALGSAHAIGSIHLNQF------------------

Phopro LIVRLDAL----PERGKCRNNSEVEVAFGTFNTLATIFVFKG------------------

Metjan FIAKVKIL---ELFKYNLAPKMKVHINIGLLTVPATIIPYKI----EKINDK--------

Metmar IVAKIKIS---DIFKYNLTPKMKVHLNVGMLIVPA-VAV---------------------

Aquaeo LIVK---------TEIDLKPGKIYQVFFGMRETVGKISVIDK------------------

Helhep IDVGIFGAP---------QHNTIYQLYLGSSKLNAKVHLIS-----EPITSH--------

Camjej CDALVK--------AKNLQNSKMI-FCVGSRQIECKINILKK------------------

Wolsuc IDVEIFPL-------EPLSHQLQVQLFIGSKKVEAKILLLEE----NPTHP---------

Sinmel LDADLSVL---ESETKPIGEWFSARFHHASAETGIRIVPLEG----P-LLPG--------

Metkan LDLKVEID---PLFPQSIGQKTMLHIHVGMRSVPARIVPHDDGFLLDSLRPG--------

Cloper VDCKFRYL---KSNEKNLVNRQRVRIYHGTSEIFGRIVLLDK----EEVKPG--------

Theten IDVKLSLL---K-EAKTLKNRERIRFYTGASEVIGRAVLLDR----DELHGG--------

Desvul WDVRLTCL---SSAARPLRNRTEVHFHHGAREVLARLHFHDR----DRLNPG--------

Geosul VDARLDYL---PSAPRELRHRSTLRLHSATYEVPAQVILLDR----DVLAPG--------

Despsy LDCEFLYI---ASNSKALKHRARVRVHLGTAEIMGRISLLDR----DELQPG--------

Symthe FAGRLHLL---ESWPKELKHGERVHLHTGTSEVLARVLLLEG----DALKPG--------

Homsap ALISVEKI---PYFRGPLQTKAKFHITVGHETVMGRLMFFSP--APDNFDQX--------

Dromel VCLQFKPI---RYYKEVIKSMRKMHISVGHNTVMANVTLFRD-------TDG--------

Caeele VLATVEPI---QFFRKSINSKSKIHVAVAFETVMAECQFLKE------------------

Pseaer -----HR---------------AFAQLVLNAPIQALHGDRLVLRDQSAQ---RTLGGGRV

Pseput -----QR---------------MFAQLLLNAPLQAVHGDRLVLRDQRAQ---RTLGGGKV

Burmal -----RR---------------ARAQLNFTEPVFAAPGDRFIVRDAQAT---RTVGGGRV

Burpse -----RR---------------ARAQLNFTEPVFAAPGDRFIVRDAQAT---RTVGGGRV

Haeinf -----DR---------------TLAEIILDSPLFLAFGDKLILRSGDTK---TLIAGARV

Pasmul -----QQ---------------GLAEIILEKPLFLAYADKLILRSGDAK---HLVAGAKV

Mansuc -----QQ---------------TFAELILDNPLFLAYGDRIILRSGDAK---QLVGGGKV

Haeduc -----EQ---------------RLAEIILDQPLHIAFNDKLMIRSGDDR---QTLAGARV

Pholum ---------------------PLLAELVLDSPLWLVEDDRLILRDISAR---KTLAGTRV

Yerpes QPLIDHQQIINNQSTSGGHSSQILAELLLDSPLWLVENDRVILRDIGAK---KTLGGARV

Yerpse QPLIGHQQIINNQSTSGGHSSQILAELLLDSPLWLVENDRVILRDIGAK---KTLGGARV

Saltyp ---------------------GALAELIFDTPLWLADNDRLVLRDISAR---ATLAGARV

Shifle ----------------------NLAELVFDTPLWLADNDRLVLRDISAR---NTLAGARV

Esccol ----------------------NLAELVFDTPLWLADNDRLVLRDISAR---NTLAGARV

Sheone -----GR---------------YLAEIVLDKPLLLCQDDPIVLRHIGGK---QTLGAGRV

Mycavi ---------------------PAAARLRLARPLPLRVADVGLLRDPGKH---RIAAGVEV

Treden -----DR---------------SLGRLSLEEPVASAWNQRAVLIRHGGS---EILASARV

Phopro ---------------------TNLARLRLKDPACCFWNQRLLLIQHGGS---NIVNSGGI

Metjan -----------------------------EEPI--------ILEE---------IKGGD-

Metmar ----------------------PFKKVTFGKSE-----ENVILNE--------VISGNE-

Aquaeo ----------------------GIYLVRLKENAIVRRGDKLVVLDSSG----NFLGGAEV

Helhep --AFGSK-------------PLTFATLKCDGIIFSIFSQHFILRDDEG-----AVLGGII

Camjej --LENDE---------------FFVHFSFDKNVFLSFDEAFILLQNN-----RVIGGGKV

Wolsuc -----NA---------------LLATLKCDTPLFALFGERFILREAG-----RTLGGGKV

Sinmel -----ER---------------RRVQLVLDRPIAAAVGDRFILRDVSAR---RTIGGGRL

Metkan -----ES---------------SYLYAKLNEPVAVREGDRTILVKLDLPPTTLRIAGSGL

Cloper -----EE---------------VYIQLRLEREICAQKGDNLVIRNYSPM---VTLGGGKI

Theten -----ES---------------CYAQIYLEDCVSVLRKDKFVIRTYSPM---LTIGGGII

Desvul -----ET---------------ALCQVRFTEPMVGVFGDRCVVRTFSPL---RTVAGGVV

Geosul -----DS---------------TFVQLRLRHPVLLLPGDPFVLRSYSPQ---ATLGGGKV

Despsy -----DE---------------TMVQVLLEEQVAVWPGDRFVVRSYSPV---VTIGGGTV

Symthe -----GT---------------AYVQLKLEEPVVVGRGDRYIIRSYSPV---HTVGGGMV

Homsap ---------------------PILDSFNFSQEYLFQ--EQYLSKDLTP-----------A

Dromel ----------------------TTSTFQLDK-------EYEYMEDVQP----AEVQHNDV

Caeele ----------------------------------KENGEFEMLPS--------LLAPCQV

Pseaer LDGAAPARNRRTPA--R---LAQLQALRQESLEE-ALPTLLGAAENGLDPQRLVQSFNRP

Pseput LDPFAPSRQRRSDA--R---LQQLQVLRDAADLEQALPALLASAPGGISPQRLERQFNRL

Burmal LDPFGPARKRRTRA--R---RAWLDALAAWLDEGRLD-ALLDEAPLGIARATLMHLTGLP

Burpse LDPFGPARKRRTRA--R---RAWLDALAAWLDEGRLD-ALLDEAPLGIARATLMHLTGLP

Haeinf LEINSPKRHKRTEV--R---LNFLANLALAENASQRIALTLQHNATTARQLMWTEQLTSL

Pasmul IEINSPKRHKRTEQ--R---LNFVYALQQAKTTTERTALYLQNKAVDAQTLMWTE----Q

Mansuc LEINSPKRHKRSEQ--R---LQWLVQLQQAHSADERIALYLQDKAVEARAITWIEQLTEL

Haeduc LEINAPKRYKRTTE--R---LIYLNQLANTHECAERIALYSQSKAVAVDRLLWLEQLCL-

Pholum INLNSPRRGKRQPA--F---LHWLTQLARTSDHAANLALHLPRGALSLDHFSWARQLTEA

Yerpes IHLAVPSRGKRQPA--Y---LAWLTALAQATSDREVLDLHLAQGPVSVRTFSWARQLTDN

Yerpse IHLAVPSRGKRQPA--Y---LAWLTALAQATSDREVLDLHLAQGPVSVRTFSWARQLTDN

Saltyp VTLNAPRRGKRKPD--Y---LHWLSTLADAQDDSAALAIHLERGAVNLPDFGWARQLNPL

Shifle VMLNPPRRGKRKPE--Y---LQWLASLARAQNDSEALADHLQRGAVNLPDF----AWARQ

Esccol VMLNPPRRGKRKPE--Y---LQWLASLARAQSDADALSVHLERGAVN----LADFAWARQ

Sheone LALKVSNRKKRTPE--R---VSKLQQLAQLTNPVNVLALLGQSEALSIDEIRWRWQLTDE

Mycavi LDVRPPELRRRGAA--R---LRA-AELATGSAPAPHIARADELRAMG-------------

Treden LASFPSY--KR---------IQFKEAFEVYRDKELP---SIHSYKFNIEGFVEDTKIKTQ

Phopro LWTGAVPKLLRNKL------YDLLMNLPESPEWR-------DYVTLNMALYGYAKK----

Metjan ------------------------------------------------------------

Metmar ------------------------------------------------------------

Aquaeo LHPKVRVTKK-----------AFIKKNIKDLLENFECYLLKERGPIGLKLEFFKRITGVS

Helhep LNPIIDPIKKHQR-------VQLLESLCKKHFDS-AFSLLCSIHKKGFGLVSSTQRFCLS

Camjej LNPLSEPLKKEQKN-------KFLMFL-KNKDFKAAFSFLKDAHKYGFGLLSSYQRFKLS

Wolsuc LNPIAESMKKRQK-------LELLNALRQ-EDFAQAFRILLSAHKRGFGIISTLQRFAIS

Sinmel LDLRAPARKRRSPE--R---LSYLQAASLSH-AGEALAALLDVPPFLVDLDVFARDRALS

Metkan VEDTS----KRE------------------------------------------------

Cloper INPVAKKAKRFKED--Y---LNELKLMEKGSIEEIVENVVLSLSETFPNSQEIIKGLGRN

Theten LDPNPEKHKRFDIE--V---IETLSNIEKLGDKFVIEKLVYESL-LPLSEEEIKRKANVV

Desvul VNPTGHDLRRRDPH--FADKVALLASLPEATEEELVQAHVDLSGNAGAGFVRLSVLTDLE

Geosul LDPMPPRRRRRSDE--A---LALLEALGERSESDTVRLLVSGSLLTGICFEDIVVRGGLS

Despsy LGNVSPRKRKRVNEADRQKNIAAYHCLKVGDYEEKILFFLKDAGMTGLCLDDIGVRLGLF

Symthe IEPHAQYRRSQAKA--A---IAELQAKESGGTGGVIAETLLRAGMTPLGLGELAKRTGVT

Homsap VTDNDEADKKAGQA-------------TEGHCPRQQWALVEFEKPVTCPRLCLVIGSRLD

Dromel IYA--------------------LLQFESPVLSPPHSTLIASKLDMDVH-----------

Caeele LFSFEKS-----------------IFLPENYSNPFMAARLEQQPGSGCRFAF--------

Pseaer RE-------HWRLPEEVVEVQTRLGPRLFAGERWFALVEQLLAGLRRFHEELPDELGPDR

Pseput RET-------WQLPDDVRVVATRQGQLLFANAQWQALKQQVLEQLARFHEQEPDQLGPDR

Burmal AQAWALP---ADAVSVAAPGKHADEARVLARGHWDALRTRVLDALAAFHQRSPDEQGPDV

Burpse AQAWALP---ADAVSVAAPGKHADEARVLARGHWDALRTRVLDALAAFHQRSPDEQGPDV

Haeinf QLD----------KALAERDAVRYQDWCFNPNYVQEKTQQILTALNIYHEQHNDQLGVSK

Pasmul LNAEQL------AEIVAVNQQVRFQDWIFNADYQQQQTEKLLTALAQYHQNHNDQLGISK

Mansuc QLN----------EIINKNNDIRFQHWCFNQNYQHRQNHKILTALSFYHDQHEDQLGLGK

Haeduc -------------SDLAEMGFDTDKKWIVSDVYKTRVQQAIVTKLTDYHQQHNDQLGVAK

Pholum TLH----------PLIVDQDMIIADGMVLSQNNAKLAKQKLLHVLAEYHQQHVDQLGLGR

Yerpes EMA----------ALLAATDTVVAGDIALSHPHAQQAQQTLLHVLALYHQQHGDQLGLGR

Yerpse EMA----------ALLAATDTVVAGDIALSHSHAQQAQQTLLHVLALYHQQHGDQLGLGR

Saltyp GMR----------QLIEQHGFIQAGDNLLSAPVAARWQRKILDTLATYHEQHRDEPGPGR

Shifle LNGEGM------RELLQQPGYIQAGYSLLNAPVAARWQRKILDTLATYHEQHRDEPGPGR

Esccol LNGEGM------RELLQQPGYIQAGYSLLNAPVAARWQRKILDTLATYHEQHRDEPGPGR

Sheone GLA----------AMLAQAGLTRIGAYGLSTQLLEQEKQQFIDLLREYHQEHPDQLGLGR

Mycavi ----------------APARGQRVGDWVVDPQWWAARRDEAAAQVRRWAGEHDIAAGMPL

Treden ELT------------SDKNEVIEYGSWRFLKKRLEFWENKILKTAQE------SQAGFT-

Phopro ------------GGLADKEQYQVCGEWLFTPECYQSSFEHIVATLE--HEKID----LSV

Metjan -----------------------------SCYCIFKLEERVVV-----------DEG---

Metmar ------------------------------CYCAFELEEKVLA-----------EVG---

Aquaeo PK-----------VANLKPESIEIRGVYYLKGFIENLKLKIKKFLDT---ELQNAFGVDK

Helhep HTQ--------SLQTASTLKDIFIDEKSLTLYPLSQVEYLKAHILEIF-LHNKSAL-LSA

Camjej HQK--------ALKLAKELNQVFVDEKNLNVYHLQSLEE-IKNFIKFILEKNPYAM-LSA

Wolsuc QQEAL-----EIAKSLPEAFVDEKELVLFPQESLQILQEEVKALL----KKNKKAL-LSP

Sinmel EAELQN----AIRSASAEVIEGSAVRHALSKRQRAAFSDEVQRVLSAFHVENPDLQGIGR

Metkan -----------------------------TFKRVSRRRGRVTRA---------DHMGKG-

Cloper IDNIDFILEKLCEDKKIIGIDNGKSKVFIHNKFFTKKSEEIYEILRKFHEENPLKVGMNK

Theten DIGK-------FSLKNLIKLKLEEKDYFYHREKYEEMCLRTKEILEDFHRKNPLKEGISK

Desvul SKRLEKV-LQALGARGRLFCFDREERAYVGADALQALSAGCLDHAGAFHAKEPLKPGMAR

Geosul ARRAEAT-MATLLSSGEVVQMVREPRIFLSRASFVSLKGGLLSEVEGYLRDNPLREGLGK

Despsy AKQLKKV-IAAPLSSRRMIMVDPSLQRYIFAEYGEQLKDRLILSLVAYHKENPLQAGIAK

Symthe VETVR-----TELPGLGADVRELEGGLWIHRRGFGQFCDAVRAELETFYGRYPLRSGLGK

Homsap A------------DIHTNTCRLAFHGILLHGLEDRNYADSFLPRLKVYKLKH--KHGLVE

Dromel ----------------STSCRLAFWGRIAWQTHSSKYFQEELPKLRIF--KRKQKVG---

Caeele --------------SGIFSQILPENGKNLPPRFTRKCRKG--------HVERVEKDGFS-

Pseaer DRLRRYALP-QLERPVFIALLDAALAGGDVHSSGPWLHLPGHEVRLSDSEEQLRARLWPL

Pseput DRLRRFVAL-PLERPAFVSLLDELLDDSAIASSGPWLHLPDHKVQLSEADSALWARLQPK

Burmal ARLRRIAAP-LADDALWRALTDALIAEGAIVRSGPWLHLPSHAVSFDAAEEALAGRLLPL

Burpse ARLRRIAAP-LADDALWRALTDALIAEGAIVRSGPWLHLPSHAVSFDAAEEALAGRLLPL

Haeinf ARLYRMATL-NQPENLIHHFIDEMLDDGRLQQTRGWIHLPEHKIQFNTEEKSRWTDVLNE

Pasmul ARLYRIATL-NQPEKLMYHFLEACLAKGQLQQTRGWLHLPEHKIQFSEDEQALWQAVLAQ

Mansuc ARLYRIAAL-NQPEKLIYHFIDELLEQGKLQQTRGWLHLAEHKIQFSTEERGLWQLVLDE

Haeduc ARLYRMAAL-AEPEQLVLALIDELIETKQLAQTRGWVHLPDHRIEFSAQELHVWQQIKPL

Pholum ARLKRMALP-TLDEALVYTLIDALLSDGQLKQLRGWLYLPEHGLSFSDEQAKLWQRIAPY

Yerpes ARLRRMALP-TLDEGVVFRLIDNLLAEGALHNTRGWLHLPEHGLAFTEQEQIGWQQAAPY

Yerpse ARLRRMALP-TLDEGVVFRLIDNLLAEGALHNTRGWLHLPEHGLAFTEQEQIGWQQAAPY

Saltyp ERLRRMALP-MEDEALVLMLIERMRDEGLIHSHHGWLHLPDHKAGFSDEQQAVWQKVEPL

Shifle ERLRRMALP-MEDEALVLLLIEKMRESGDILSHHGWLHLPDHKAGFSEEQQAIWQKAEPL

Esccol ERLRRMALP-MEDEALVLLLIEKMRESGDIHSHHGWLHLPDHKAGFSEEQQAIWQKAEPL

Sheone NRLQRMSHS-VLPSELANKVLDSLCQEGQMALRGALLQLADHRIVLDSEAQQCWQRLAPW

Mycavi ETLRRQVG--LPAAELVPKLLQGTGFE----VADGLVRAPGAGLPARVDQAV--REVEAW

Treden --LEEVDLP--VPQRVKTTVLKKLCDEKKLEKEAHVYKLSGR----TGDDISKNAKALLD

Phopro NELSGKV---QMSVPVLSDLLKQLVADGKLRQNDDLYSLGN-----GRNEAELSSIAKQI

Metjan -------------DKILIMRLD---------LPPTTLRICGFGEVIDFGEVEVKKIVVKE

Metmar -------------DRVLITRLD---------LPPTTLRICGHGL-IEEFKPINDLNIKKE

Aquaeo EKVKSMF---SLNEELLKYILDELKTYKIV----NELIIDERKSDLEKNED--FQKLMSI

Helhep QSLCVKIK--WASLGLCQKALDELLNEKQI-RYKNGLYL-SQWCQIKDIKTYVQERILEI

Camjej HSLALRIT--WASENFCELGLKEM--SNLLD-FQNGIYFKK-GIDFEKLQEKNNNQMYEI

Wolsuc ASLGLKAP--WASEALLSLSLARLEQEGVITLEGGLYLAPDSGIV--SIEEYLEETLYKI

Sinmel ERLRLQVTP-RLPPPAFLVALRAEQTAGRLVLEGAFVRLPGHEVRLSEKEEELYARILPH

Metkan --L---------------AVVDGLALN------------KEH------AERLVGEKVR--

Cloper EELRSKSFSNKIKQKIFQSFLELMKKKEIIKEGINIISLKDFEIKLTKAQKAIREKILKA

Theten EELKNKLFG-DIKSKLCDFIFELMEKEEIIKIKNQLVALKDFKVVLNEEQEKLKERIIKI

Desvul GVLASGWGR-GLHPRLIHFVVERLIKQGALVAEGDVLRLPGHKVSLASDQEGLRGKLLAI

Geosul EELKTRLPR-RSDPRFFTPLLTSLEKEGKIVVDRDLVKLPGRKGTITVDQADLQVRLEKA

Despsy EELRSGLGA-RVDAKLFNWCLADLVRKDVLVQEDSLVHMASHEVALKADEEELQRDLLVW

Symthe EELRRKL---GLGGKEFAALLAAAEAAGELSVNRDRVALPGRAPALNGAQAKLAERLRAA

Homsap RAMD--------DYSVIGRSLFKKETNIQL-----FVGLKVH---LSTGELGI---IDSA

Dromel -SIQRVV---NSSEVIVQNLFKDAKRD---------LYV-GKNVELSTGEKG---RIERT

Caeele --------------AICTGMFKA-------------------ETNFD---------VFRG

Pseaer LE-AGRYDPPWVRDLARELR-QDEAVMRNLLRKLARLGQLQQVVRDLFYPEATLRQLAEI

Pseput LI-AGQFDPPWVRTLASEEN-CAEADVRLLLRKLARLGLVHQVVRDLFYPEATLQRMAAL

Burmal VA-AGRYDPPWVRDHAAATL-MAEDAVRALLRKLARRGDVHQVVRDLFYHRDVIAELARL

Burpse VA-AGRYDPPWVRDHAAATL-MAEDAVRALLRKLARRGDVHQVVRDLFYHRDVIAELARL

Haeinf FE-KANGQAIWVRDMANALA-IDESIMRNFMYKAGKLGYLTPIVKDRFFLTETLYAYARL

Pasmul FE-QQHGHPLWVRDLANVLG-QDESAMRNFLYKAGKLGYLTPIVKDRFFLTETLYAYSRL

Mansuc FE-KQKGQPLWVRDLAQNLG-FDETLMRNFLYKAGKLGFLTAVVKDRFFLTEHIYGYARL

Haeduc F--EAKREALWVRDVANTLL-IDETVMRDLLYKAGKLGYLMPIVKDRFFLHEHIVEFAQI

Pholum FT-D---DPWWVRDLTADLK-EEEATVRSLLKKAAQLGHIIAIVPDRYYCSQRIQQFADL

Yerpes FV-D---DPWWVRDLAAELQ-AAEGEMRSLLRKAAQLGYITAVVTDRYYLSQRIEQLADL

Yerpse FV-D---DPWWVRDLAAELQ-AAEGEMRSLLRKAAQLGYITAVVTDRYYLSQRIEQLADL

Saltyp F----GDEPWWVRDLAKETG-TEEQLMRLVLRQAAQQGIITAIVKDRYYRNDRIVAFANM

Shifle F----GDEPWWVRDLAKETG-TDEQAMRLTLRQAAQQGIITAIVKDRYYRNDRIVEFANM

Esccol F----GDEPWWVRDLAKETG-TDEQAMRLTLRQAAQQGIITAIVKDRYYRNDRIVEFANM

Sheone LA--QQTSPVWVTEMGEYLD-LEPAKLRLLCFQLVQQGFITAVVKDRYLLSEQLCHYANL

Mycavi LAAEPFRAPE--ADELAGLG-----LGPRELAAAVRTGRLTRIADGVVLGPDALARAAGV

Treden AAFKAGFEGIEI-DKIKL------PQARKEARDLIKLGKLICLENFLHYHTDFYKKAVNS

Phopro LNKMVEAQKQGFEAEKEKL-----PGAQKELRNLVRLGFAVPLEGKIYYEKNLYDSLVTG

Metjan GKVVKKKDKIYIEGLASS-----KTAGEKLIG-----GKV-------YIPDKNIWGV---

Metmar VLREGKVK-------------IDKG--RTVIDGLAQS----KVAADKLIGEEI-------

Aquaeo LKGG-------IKEEREIIL---EGIPKEILTLSIKRKYAHRIGEYLIISDELLKKYINE

Helhep LA-QGHYAPPAPYNIYEELD-IDKKVGDDALKALTQAHKVVRITHNLFITSSALNHIVSL

Camjej LK-KQGIKPEAPYNLYDFLE-LDRKSGDNILKKLTQKGLVVRLSHNLFIEKQALEKLMQE

Wolsuc LE-KQGVAPEAPYNLYDSLD-IDRKSGDNALKRLTSAQKVIRLAHNLFITSESLRGVLEL

Sinmel LEGEERFRPPRVRDFAEALG-VDEREIRRILKLCARLGRVDQIRHDHFFTRQTTAEMVAI

Metkan ----------------------------------TEGGVEGKIVDTHGTRGAVLVDFEGE

Cloper YL-ESGIVSPKIKDIIEDKK-LEKEYIK-IYNLLIEEGTLIKLPEDVVMHKEVLQSVKEK

Theten YR-DSKFEPPKLSELEGY------PEIIPMVEYLVNIGELVKLDEEIFLSKDNYEEAQNV

Desvul YS-EAGMTPPNLKDVLETLS-LEQKEAAPVLKLLHDGGELVRVKDGLLFHRSALDELKGR

Geosul LQ-RGGYEPPTIKEMCDSLR-CAEKMLLEHLNILAREERAVKVKSDIFYAPEPVADIREK

Despsy YQ-QRGTATPTIRETMDHFADYSESLVKEVLALQLREGKLVKISETLYYAKEIIDELSRQ

Symthe IA-GAGFSPLTVAELKQQVP---GADTEEVIIYLIEQGEAVRVGDDLVIAAPVLEEAVRR

Homsap FGQSGKFK----IHIPGGLSPESKKILTPALKKRARAGRGEATRQEE--SAERSEPSQHV

Dromel F------------------------------------GQTSKVA---ITFQDALSPE---

Caeele FQICTSSGP---------------------------RGKI--------------------

Pseaer AVQVR--DPAGL-IRPAEFRDRL-GIGRKRSIQILEHFDRIGLTRRI--GNDRRLREGSA

Pseput MLGQA--SETQI-VQVAAFRDML-GIGRKRSVQILEYFDRIGLTRRV--ADQRHIRADSA

Burmal IARLA--GEHGGGLDAATFRDAT-GLGRKRAIQILEFFDRVGYTRFH--RDLHWLRADSR

Burpse IARLA--GEHGGGLDAATFRDAT-GLGRKRAIQILEFFDRVGYTRFH--RDLHWLRADSR

Haeinf IKQIA--EEKGK-VSVNEVRDKL-NFGRKLTVQLMEYFDRMGFLRRK--GNDHILRDKNV

Pasmul VKQFI--AEHGA-ISVNQLRDEL-QFGRKLVVQLIEYFDRCGFLRRK--GNIHVLRDSDV

Mansuc IKQMI--EQNGA-VSVNQLRDEL-QIGRKLAVQLMEYFDRSGYLRRK--GNIHILRDTEA

Haeduc IKQFI--ANHGA-ISVNQLRDQL-GFGRKMTVQLIEYFDRCGFLRRK--SNLHLLRDADI

Pholum IRQFN--QDHGA-ITAADFRDQL-GIGRKLAIQILEFFDRSGFTRRQ--AESHILRDNGL

Yerpes VRELD--SRQGS-VSAADFRDRL-GIGRKLAIQILEFFDRSGFTRRR--GNGHILRDSGL

Yerpse VRELD--SSQGS-VSAADFRDRL-GIGRKLAIQILEFFDRSGFTRRR--GNGHILRDSGL

Saltyp IRELD--QERGS-TCAADFRDRL-NVGRKLAIQILEYFDRIGFTRRR--GNDHLLRDALL

Shifle IRDLD--QECGS-TCAADFRDRL-GVGRKLAIQILEYFDRIGFTRRR--GNDHLLRDALL

Esccol IRDLD--QECGS-TCAADFRDRL-GVGRKLAIQILEYFDRIGFTRRR--GNDHLLRDALL

Sheone VREHV--DTHGK-LETAEFKDMI-GLGRKVSIQLLEFFDRSGFTRRKFRSNSREIRDGEL

Mycavi LAGL-----PQP-FTVSEARRAL-GTTRRVAVPLLEQLDARRVTRRA--GDGTRVLIAD-

Treden LFAKY--KTGDK-ISIADAREVT-GLSRKYILPILNLLEKEGKLKRQ--DNDRIVL----

Phopro IIQGR--QIDDR-FDIAEARECA-GLSRKYMIPVLNRMESDGWIKRD--NNVRQVLRLQ-

Metjan ------------------IKGT---FGTKGAL-IAE-FDEEVNG-----GEKVVLKRVRK

Metmar -------SIDGKDV-VGKIKGT---FGTKGLLT-AEF---SGTVENR---DKVILNRLRR

Aquaeo LKEL------GKTFNVQQAKNKL-GLTRKYLIPLLEYLDYLGLTVRE--GNERRWKR---

Helhep MREII--STHSY-VDVNLLRKRT-DLSRKYLIGYLEYLDKFDDIECN--ENKRSFKYGT-

Camjej CLNLL--KNQ-S-LDVQSMKEYF-NLSRKYAIAYLEYLDKFPQVSKE--AEKRFLTNI--

Wolsuc MRRII--KEEGY-IEINNLKAHL-PLSRKYLIAYLEYLDLFSDIRKE--ENKRVFR----

Sinmel IRQVAANAERGE-FSAGLFRDRV-NNGRKVAIEILEFFDRQGVTIRH--GDVRRVNPHRL

Metkan VK-------TGERVVLERVRDV------K--------IDL--------------------

Cloper IIEWL--NKNGS-ITLGETKEIL-GVSRKYLVAILEYLDNEEITKRV--EDKRVLN----

Theten LINYL--KENKE-ITLAVYRDLL-NTSRKYAMAILEYFDSIKLTKRI--GDVRVLAREIK

Desvul VARWF--DDHDD-LDPSGFKELSGGLSRKYIIPLLEYFDRERVTIRV--GDKRQLRGAKA

Geosul LMAYL--REKGE-IMPPEFRELT-GLSRKFMIPLLEFFDQEKITIRV--GDKRVLRRG--

Despsy IEERI--QRLGE-IDAPGMKELS-GLSRKFSIPILEYFDRVKLTMRV--GDKRILRRKA-

Symthe TREHL--KARGR-LTVAEFRDLL-GTSRKYALPLLEWMDEQRITRRA--GEERVAGPNG-

Homsap VFSL-------------TFK-------R--YV-----FDT----------HKRMVQSP--

Dromel --------------TISNVKNV------KVLLNCKKYV-----------FN----KQAGL

Caeele ---------EGAFGKSGKFRVT---FAEKIDGILTEKEEISLFLKKY--HNENRLVSYVP

:

Pseaer LAEQLLGGVSP-------------------------------------

Pseput LAQQHARH----------------------------------------

Burmal LLAGSR------------------------------------------

Burpse LLAGSR------------------------------------------

Haeinf FDL---------------------------------------------

Pasmul FDL---------------------------------------------

Mansuc FDL---------------------------------------------

Haeduc FSSSTH------------------------------------------

Pholum F-----------------------------------------------

Yerpes FSATGS------------------------------------------

Yerpse FSATGS------------------------------------------

Saltyp FPQKE-------------------------------------------

Shifle FPEK--------------------------------------------

Esccol FPEK--------------------------------------------

Sheone FYSHENWRKNIGVEPTQDCWQPYPDLKSGRLTGDDVLPIEDNLIVENV

Mycavi ------------------------------------------------

Treden ------------------------------------------------

Phopro ------------------------------------------------

Metjan WG----------------------------------------------

Metmar WG----------------------------------------------

Aquaeo ------------------------------------------------

Helhep ------------------------------------------------

Camjej ------------------------------------------------

Wolsuc ------------------------------------------------

Sinmel DLYEGPVPEADEGRGSSPVERPDFKFYRAGS-----------------

Metkan ------------------------------------------------

Cloper ------------------------------------------------

Theten N-----------------------------------------------

Desvul G-----------------------------------------------

Geosul ------------------------------------------------

Despsy ------------------------------------------------

Symthe ------------------------------------------------

Homsap ------------------------------------------------

Dromel FQ----------------------------------------------

Caeele D-----------------------------------------------

**SELC**

CLUSTAL FORMAT for T-COFFEE Version_1.37, CPU=0.00 sec, SCORE=27, Nseq=35, Len=105

Geosul ---GGAGGTACGTCGGTCGCTGGT-GGGCCGCCCGGTCTTCAAAACCGGTGTGGGGGT-T

Despsy ----GAAGTGGACGGAGCACTGGT-GGCTCCCCCGGATTTCAAATCCGGTGTAACGGGTT

Aquaeo ---GGGAGAGGTTGGCCGGCTGGT-GCCGCCCCGGGACTTCAAATCCCGTGGGAGGTCCC

Cloper ----GGAGTAGATAGGG-GCTGGT-GTCTCTACAGGTCTTCAAAACCTAGTTATGG---T

Theten ---GGGAGTAGATAGGC-GCTGGT-GTGCCTCCTAGACTTCAAATCTAC-----GGTCTC

Symthe ---GGGAGTAGATCGGGTCCTGGT-GGCCCGCACGGTCTTCAAAACCGCTT--GAGCCGT

Mycavi ---GGAGGCGTGTC-CGGTCTGGT-GACCGGCGCGGTCTTCAAAACCGT-----GGAACG

Treden ---GGAAGCAGATGGGG-TCTGGT-GGCTCCCGCGGTCTTCAAAACCGT----AGGT--T

Phopro ---GGAAGTGGATGGGG-TCTGGT-GGCCCTCCCGGTCTTCAAAACCGAC---GTGA---

Pholum ---GGAAGATCATCGTC-CCCGGT-GGGGCGGCTGGACTTCAAATCCAGAT-GGGGCC--

Yerpes ---GGAAGATCATCGTC-CCCGGT-GGGGCGGCTGGACTTCAAATCCAGAT-GGGGCC--

Yerpse ---GGAAGATCATCGTC-CCCGGT-GGGGCGGCTGGACTTCAAATCCAGAT-GGGGCC--

Esccol ---GGAAGATCGTCGTC-TCCGGT-GAGGCGGCTGGACTTCAAATCCAGTT-GGGGCC--

Saltyp ---GGAAGATCGTCGTC-TCCGGT-GAGGCGGCTGGACTTCAAATCCAGTT-GGGGCC--

Shifle ---GGAAGATCGTCGTC-TCCGGT-GAGGCGGCTGGACTTCAAATCCAGTT-GGGGCC--

Sheone ---GGAAGAACGTCGTC-TCCGGT-GAGGCGTCCGGATTTCAAATCCGGGT-AGGGC--T

Mansuc ---GGAAGGTCGTCGTG-TCCGGT-GATGCGGCAGGACTTCAAATCCTGTT-AGGGC--T

Haeduc ---GGAAGTGAGTCGTC-TCCGGT-GAGACGGCAGGATTTCAAATCCTGTT-AAGGAC--

Haeinf ---GGAGGGGCGTCATC-TCCGGT-GAGGTGGCAGGATTTCAAATCCTGTT-GAGGAC--

Pasmul ---GGAGGAGCGTCGTT-TCCGGT-GAAGCGGCAGGACTTCAAATCCTGTT-GAGGT--T

Sinmel ---GGAAGAGAATCATG-CCCGGT-GGCATGTCCGGACTTCAAATCCGGGT-GGGGC--A

Pseaer ---GGAAGGCAATCA-CGCCCGGT-GGCGTGGCCGGACTTCAAATCCGGTG-GGGGAC-G

Pseput ---GGAAGGCAATCG-CGCCCGGT-GGCGCGGCCGGGCTTCAAACCCGGTT-GGGGAC-G

Burpse ----GAAGGCATTCGTA-TCCGGT-GGTGCGGCTGGGCTTCAAACCCAGTT-GGTGC--G

Burmal ---GGAAGGCATTCGTA-TCCGGT-GGTGCGGCTGGGCTTCAAACCCAGTT-GGTGC--G

Desvul ---GGAAGCGTTTTCTA-TCCGGT-GATAGGCCCGGTCTTCAAAACCGGTGGTAGGTC--

Camjej ---GGAAGATTAGCGTA-TCTGGT-GATCGCCACTGACTTCAAATCAGATGAAAGGA-TA

Wolsuc ---GGGGGGCATGCGAC-CCTGGT-GGGCGCCTCGGGCTTCAAACCCGCTGGATGGGCAG

Helhep --AGGAGAGCAAACGTC-TCTGGT-GGGCGTCTTGGGCTTCAAACCCAT-TGAAGGATTA

Metjan CGCGGG-GTAC--CGGGCT-TGGTAGCCCGGGGCGGACTTCAGATCCGTC--GAGG----

Metmar --GGCACGGGGTGCTTATCTTGGTAGATGAGGGCGGACTTCAGATCCG--TCGA-GTTCC

Metkan ---GGCCGCCGCCACCGGGGTGGTC-CCCGGGCCGGACTTCAGATCCGGC---GCGCCCC

Homsap GCTCG--GATGATCCTCAG-TGGT-CTGGGGTGCAGGCTTCAAACCTG--TAGCTGTCTA

Caeele GCCCG--GATGAACCATGGC-GGTCTGTG-GTGCAGACTTCAAATCTG--T--AGGC--G

Dromel GCC--CCACTGAACTTCGG-TGGTCCGGG-GTGCGGACTTCAAATCCG--TAGTCGATTT

*** * **** * * *

Geosul GAGAAAACT--CCCAGGTGGGTTCGATTCCCATGTACCTCC----

Despsy AATAGCTCG---TTAGGTGGGTTCGATTCCCATGCACTTCCG---

Aquaeo GCAAGGGAG--CTCCGGAGGGTTCGATTCCCTCCCTCTCCC----

Cloper GCTAATACCATCATGGGTGGGTTCGATTCCCACA-TATTCC----

Theten GCTATTTAAGCGAGAGGTGGGTTCGATTCCCACATACTCCCG---

Symthe AGTGATCCTGCGGCTGGTGGGTTCGATTCCCACATACTCCCGCC-

Mycavi GCAGACGCTGCCGCTGGCGGGTTCGATTCCCGTCCGCCTCC----

Treden GCATAATTCG-CAATGGCAGGTTCGATTCCTGCC-TCTTCC----

Phopro GCTGAATAGG-CTTTGGCAGGTTCGATTCCTGCTC-CTTCC----

Pholum GCCAG--CGGTCTCTGGCAGGTTCGACTCCTGTGATCTTCC----

Yerpes GCCAG--CGGTCCCTGGCAGGTTCGACTCCTGTGATCTTCC----

Yerpse GCCAG--CGGTCCCTGGCAGGTTCGACTCCTGTGATCTTCC----

Esccol GCCAG--CGGTCCCGGGCAGGTTCGACTCCTGTGATCTTCC----

Saltyp GCCAG--CGGTCCCGGGCAGGTTCGACTCCTGTGATCTTCC----

Shifle GCCAG--CGGTCCCGGGCAGGTTCGACTCCTGTGATCTTCC----

Sheone GCCAG--CAGTCCTGGGTAGGTTCGACTCCTATGTTCTTCC----

Mansuc GCCAG--CAGTCCTGGGTGGGTTCGACTCCCATGACCTTCC----

Haeduc GCCAG--CGTGCTTGGGTGGGTTCGACTCCCATTCACTTCC----

Haeinf GCCAG--CGTTCTTGGGTGGGTTCAACTCCCATTCCCCTCC----

Pasmul GCCAG--CAATCTCGGGTGGGTTCGACTCCCATTCTCCTCC----

Sinmel GCGAG-GCTGCCTTGGGTGGGTTCGACTCCCATTCTCTTCC----

Pseaer GC-AGC-CGTTCCTGGGTGGGTTCGACTCCCACTGCCTTCC----

Pseput GC-AGC-CGTTCCCGGGCAGGTTCGACTCCCGCTGCCTTCCGCCA

Burpse GTCAGCCCGCGC-CAGGTCGGTTCGACTCCGGCTGCCTTCCGCCA

Burmal GTCAGCCCGCGC-CAGGTCGGTTCGACTCCGGCTGCCTTCCGCCA

Desvul GAGAG----GTCTACGGTAGGTTCGACTCCTATACGCTTCC----

Camjej G-TTGACTATTCTTTGGGGAGTTCGATTCTCTCATCTTCTCGCCA

Wolsuc G--TGTCTGTCTGTCGAGAAGTTCGATTCTTCTGC-CTTCTC---

Helhep GGTGTCTAATTCTTGGG-GAGTTCGATTCTCTCGCTCT-CCTG--

Metjan GCGAGAGC---CCTCG--GGGTTCGATTCCCCCC--CTGCGC---

Metmar GTTGG--AATTC---G--GGGTTCGATTCCCCCCCTGCGCCGTTT

Metkan GAGTG---GGGCGC-G--GGGTTCAATTCCCCGCGGCGGCCGCG-

Homsap GTGA-------C--AGAGTGGTTCAATTCC-AC---CTTTGTAGG

Caeele GTTAGCGC---CGCAG--TGGTTCGACTCC-AC---CTTTCGGGT

Dromel GC-------GTCGAAG--TGGTTCGATTCCA-C---CTGGGGGGC

* **** * **

**SELD**

CLUSTAL FORMAT for T-COFFEE Version_1.37, CPU=0.00 sec, SCORE=43, Nseq=42, Len=460

Metjan -----------------------------------MERGNEKIKLTELVKLHGCACKLPS

Metmar ------------------------------------------------------------

Metkan --------------------------------------MSRKKSLVEMADLHGCACKLPQ

Homsap -------------------------MSTRESFNPESYELDKSFRLTRFTELKGTGCKVPQ

Dromel -----MSYAADVLNSAHLELHGGGDAELRRPFDPTAHDLDASFRLTRFADLKGRGCKVPQ

Caeele ---------------------MNRIERILEGFDPVSNGLDEDFVLTKLTGMKGCGCKVPR

Aquaeo --------------------------------------------L------KG-------

Cloper ------------------------------------------------------------

Camjej ------------------------------------------------------------

Helhep ------------------------------------------------------------

Wolsuc MPLKSNSQHSKSAILSRFIHFCDNLLCKSKRSESNANAMNETTKITQFVKVAGCAGKVGP

Entfae -----------------------------------------MDFLSQCT-SGGCGAKIES

Porgin -------------------------------------MNRSSFDLLSTVEYGGCSAKLDP

Mycavi --------------------------------------------VVR-----GLGA-ASP

Symthe ------------------------------------------------------------

Bdebac -------------------------------------MTSTKISLTQTVQKGGCAAKVAA

Niteur ------LPWAGTAGVFDTSTSFPIPSSSQERFLFRTIMPSEKIALTQTVQKGGCAAKVAA

Theten ------------------------------------------------------------

Geosul --------------------------------------MTEGIKLTSLVKAAGCAAKLGP

Treden ---------------------------------MSCSLINEDFDLLKAAKNPGCGAKLSA

Despsy ------------------------------------------------------------

Desvul ---------------------------------------MTVDRLTSRSRAAGCAAKIAP

Phopro2 ------------------------------------------------------------

Haeinf --------------------------------------MEEKIRLTQYSHGAGCGCKISP

Pasmul -------------------MQAKIEQNLTALEQEMNDTIASTVRLTQYSHGAGCGCKISP

Mansuc ------------------------------------------------------------

Yerpes -------------------------------------MASPAIRLTQYSHGAGCGCKISP

Yerpse -------------------------------------MASPAIRLTQYSHGAGCGCKISP

Pholum --------------------------------------MSTEVRLTQYSHGAGCGCKISP

Saltyp -------------------------------------MSKQAIRLTQYSHGAGCGCKISP

Shifle -----------------------------------MSMSENSIRLTQYSHGAGCGCKISP

Esccol -------------------------------------MSENSIRLTQYSHGAGCGCKISP

Phopro1 ---------------------------------------MENVRLTQYSHGAGCGCKISP

Pseput --------------------------------------MSEPIRLTQYSHGAGCGCKISP

Psesyr --------------------------------------MNEPIRLTQYSHGAGCGCKISP

Pseaer --------------------------------------MSEAIRLTQYSHGAGCGCKISP

Borbro --------------------------------------MNTPVRLTQYSHGAGCGCKISP

Sheone -------------------------------MSNSTLPSTDSIKLTEYSHGAGCGCKISP

Haeduc ------------------------------------------------------------

Burpse -------------------------------MTETKQTDAAAPRLTSLSHGGGCGCKIAP

Burmal -------------------------------MTETKQTDAAAPRLTSLSHGGGCGCKIAP

Sinmel -------------------------------------MNTLAPRLTDLAHGGGCGCKLAP

Metjan TELEFLVKGIV-TDDDLLD-----KNILVG----LGDDASII--KRNGLVIAKTVDVFTP

Metmar ----------------------------VG----LGDDAAVV--IKNGMAIVKTIDVFTP

Metkan GDLEDLLKGVE---LPEEG-----GRVEVG----VGDDAAVI-RV-DGGYVIQSVDFFTP

Homsap DVLQKLLESLQENHFQEDEQFLGAVMPRLG--IGM--DTCVIPLRHGGLSLVQTTDYIYP

Dromel DVLSKLVSALQQ-DYSAQDQEPQFLNVAIP-RIGIGLDCSVIPLRHGGLCLVQTTDFFYP

Caeele NVLLQLLQTFKTDLVINND------EVDIGL------DSCVIPLRHPGLRLVQTTDFFYP

Aquaeo ---FNI----------YTD-----ESTLV----SIGDDAGVY--EHNGIIWVYTVDIITP

Cloper ------------------------------------------------------------

Camjej --MQK------------------TPALLSD--IGNNEDASVY-QISPDLALVQTLDFITP

Helhep ---------------------------------------MLY-APNDEYAMLSSVDFITP

Wolsuc GDLHH-LSSLL---HQPHD-----PSLLVG--FEGNEDAGVY-QISPECALVQTADFITP

Entfae RVLNQLLQHLP---ETPAN-----ERLLVG--FEHADDAAVY-QITSEKALISTVDFFSP

Porgin AKLSELLHDIP----LPVD-----SRIMVD--VSTHDDAGVY-RLNDDTALIVTTDFFPP

Mycavi R--DPL------------------GELLVG--LDSGDDAAVV-RIRQGTALIATTDFFTP

Symthe -----MLRHLP---PSLTN-----ADVIVG--LDTPDDAGVY-RITDDLALVQTADFFTP

Bdebac SELREILKQVK---FPAAH-----PALMVD--GGLFDDAAIY-KINDEIALVQTLDFFTP

Niteur TELRRILQQVG---FPAAH-----PALMVD--GRYFDDAAIY-RVNEETALVQTLDFFTP

Theten --LAQVLCQL----EITWN-----ENLLVG--LNTNDDAAVY-RLNEDIAIVHTVDYFTP

Geosul AGLEQALSDM----VREDD-----PNLLVG--PETADDAGVY-RIGEGLALVETVDIITP

Treden GALDKLLKNF----SVRND-----DNLLVG--FNTSDDAAVY-KINDKTALISTIDFFPP

Despsy --------------------------MLVG--MATADDAGVY-KVSDELALIQTLDFFTP

Desvul GDLERILATLPR--DPREG-----ERVVVG--TRDNEDAAIV-RVPGGKAIVQTLDFFTP

Phopro2 --MAQVLQTISP-LFPEQD----YPNLMVG--LAVSDDAAVY-KINDDVAVIQTLDFFTP

Haeinf KVLGTILHSEL---EKFYD-----PNLIVG--NETADDAAVY-DLGNGTAIISTTDFFMP

Pasmul KVLEKILHSDM---EKWVD-----PHLLVG--NETKDDAAVY-DIGNGIGIISTTDFFMP

Mansuc -MLGTILHSQL---EQFVD-----PHLLVG--NDTNDDAAVY-DIGNGTCIISTTDFFMP

Yerpes KVLDKILHTEQ---QKFFD-----PRLLVG--NETRDDAAVY-DIGNGVGIISTTDFFMP

Yerpse KVLDKILHTEQ---QKFFD-----PRLLVG--NETRDDAAVY-DIGNGVGIISTTDFFMP

Pholum KVLETILHSEQ---EKFLD-----PHLLVG--NETRDDAAVY-DIGNGTGIISTTDFFMP

Saltyp KVLETILHSEQ---AKFVD-----PNLLVG--NETRDDAAVY-DLGNGTSIISTTDFFMP

Shifle KVLETILHSEQ---AKFVD-----PNLLVG--NETRDDAAVY-DLGNGTSVISTTDFFMP

Esccol KVLETILHSEQ---AKFVD-----PNLLVG--NETRDDAAVY-DLGNGTSVISTTDFFMP

Phopro1 QVLDTILRTQL---APFSD-----PNLLVG--NESKDDAAVY-DLGNGTAAISTTDFFMP

Pseput KVLDVILAESG---TQALD-----PKLWVG--NASRDDAAVY-ALDDERGVVSTTDFFMP

Psesyr KVLDVILAGSG---AQNLD-----PKLWVG--NTSRDDAAVY-ALDDERGVVSTTDFFMP

Pseaer KVLEVILAGSG---AQNLD-----PKLWVG--NASRDDAAVY-ALDEERGVVSTTDFFMP

Borbro KVLEIILAGSG---AQNLD-----PRLWVG--NASRDDAAVY-ALDDQRGVVSTTDFFMP

Sheone KVLTTILASQL---PVFTD-----PNLLVG--NQSRDDAAVY-KLNEEVGIISTTDFFMP

Haeduc -VLGTILQSEL---DKFVD-----PKLLVG--NETADDAAVY-DIGNGLAIISTTDFFMP

Burpse GVLSELLRRTA---PPALF-----PDLLVG--TETSDDAAVY-RLNDEQAIVATTDFFMP

Burmal GVLSELLRRTA---PPALF-----PDLLVG--TETSDDAAVY-RLNDEQAIVATTDFFMP

Sinmel SVLQQLLANQP---AARPF-----AQLLVG--NDMADDAAVW-QVDDNTCVIATTDFFMP

Metjan IVDDPYIQGKIAACNSTSDIYAMGLLDIVGVLAIVGIP-EKLPI----HVVREMLKGFQD

Metmar IVDDPYLQGRIAACNSTSDVYAMGISEIIGGLVFLGIP-PELPV----PVAKKMLQGFQD

Metkan IHPDPYTQGRIAANNSINDVFAMGATEVLSVLVVSGFP-RELPE----EDAREMLQGFAD

Homsap IVDDPYMMGRIACANVLSDLYAMGVTECDNMLMLLGV-SNKMTDRERDKVMPLIIQGFKD

Dromel IVDDPYMMGKIACANVLSDLYAMGVTDCDNMLMLLAV-STKMTEKERDVVIPLIMRGFKD

Caeele LIDDPYIMGRVTCANVLSDLYAMGVSECDNMLMLLAVAID-LNEKQRDIVVPLFIQGFKD

Aquaeo VVNDPYLWGAISTANALSDVYAMGGIPVNAL-AISCFNNCELDI----EIFREVIRGALD

Cloper ----------------------------VAL-NIVCFP-NCLNM----NILGEILRGGAD

Camjej IVDSAYHFGAIAAANALSDVFAMGAEVINAL-NIVGFDTCNHDL----NILKELLEGAND

Helhep VVDDPYLYGQIAAANALSDIFAMGGEVKSAL-NLLMWDNTHFDS----AVANAILKGGLN

Wolsuc VVNDPYLYGQIAAANSLSDVFAMGGRVKTAL-NLLMWDKCHLDE----KMISEVLQGGLS

Entfae MVNDPLSFGKIAVANALSDVYAMGGTVLYAL-NLVCFPQ-KMSK----EWLEQMLLGGSL

Porgin VCSDPYTFGRIAAANALSDVYAMGGRPLLVL-NLTMFPSEGIPV----EVLADILRGGQQ

Mycavi VVDDAYDWGRIAATNALSDVYAMGGRPVVAV-NLLGWPREVLPF----ELAAETLRGGLD

Symthe IVDDPYWFGAIAAANSLSDIYAMGGRPVTAL-NLVSFPVSKLDK----RILAEILRGGAE

Bdebac IVDTPKLFGEIAAANALSDVYAMGGKPKTAM-GILAFPLATLPK----EVIVDVMQGASD

Niteur IVDTPKLFGEIAAANALSDVYAMGGRPVTAM-GILAFPLAALPE----QVIVDVLQGASD

Theten VVDDPYDFGQIAAANALSDVYAMGAVPLFAL-NVVCFPAAYI------DVLKEVLRGGND

Geosul LVDDPYTFGRIAAANALSDVFAMGGRPVTAM-NLVFFPACALPG----SVLSAILAGGHD

Treden VSGDPYIFGQVAAANSLSDIYAMGGEPKLAL-NLFCITK-DMPE----DMIKEILRGGFD

Despsy IVDDPYDFGRIAAANALSDVYAMGGVPQTAM-NIVAYPMKTLGR----EPLREMLAGGSA

Desvul IVDDPYLFGQIAAANALSDVYAMGGEPWCAL-NIVCFPVKELPE----DILADILRGGAD

Phopro2 IVDDPYDFGAIAAANALSDVYAMGGQVTLAM-NIFCVPVD-LPQ----EVVGQILKGGAD

Haeinf IVDDPFDFGRIAATNAISDIFAMGGKPIMGI-AILGFPTNVLPA----EVAQKIVDGGRF

Pasmul IVDDPFDFGRIAATNAISDIFAMGGKPIMAI-AILGFPIKLLPP----EVAQRIVDGGRF

Mansuc IVDDPFDFGRIAATNAISDIFAMGGKPIMAI-AILGFPINVLPA----EVAQKIVDGGRF

Yerpes IVDDPFDFGRIAATNAISDVYAMGGKPIMAI-AILGWPIDKLAP----EIAQQVIEGGRY

Yerpse IVDDPFDFGRIAATNAISDVYAMGGKPIMAI-AILGWPIDKLAP----EIAQQVIEGGRY

Pholum IVDDPFDFGRIAATNAISDIYAMGGKPIMAI-AILGWPIDKLAP----EIARKVIEGGRA

Saltyp IVDNPFDFGRIAATNAISDIFAMGGKPIMAI-AILGWPINTLSP----DIAREVTEGGRF

Shifle IVDNPFDFGRIAATNAISDIFAMGGKPIMAI-AILGWPINKLSP----EIAREVTEGGRY

Esccol IVDNPFDFGRIAATNAISDIFAMGGKPIMAI-AILGWPINKLSP----EIAREVTEGGRY

Phopro1 IVDDPFDFGRIAATNAISDIYAMGGKPIMAI-AILGWPVNVLAP----EIAQQVIEGGRS

Pseput IVDDPYDFGRIAATNAISDIYAMGGDPLMAI-AILGWPVNVLPP----EVAREVIRGGRA

Psesyr IVDDPFDFGRIAATNAISDIYAMGGDPLMAI-AILGWPVNVLPP----EVAREVIRGGRA

Pseaer IVDDPFDFGRIAATNAISDIYAMGGDPLMAI-AILGWPVNVLAA----EVAREVIAGGRK

Borbro IVDDPFDFGRIAATNAISDIYAMGGDPLLAI-AILGWPVNVLPP----EVAREVVRGGRA

Sheone IVDDPFTFGRIAATNAISDIYAMGGTPMMAI-AILGWPINKLPA----EIAQQVVDGGRQ

Haeduc IVDDPFDFGRIAATNALSDIFAMGGKPLMAI-AILGFPIDKLPA----EVAQKIIEGGRF

Burpse IVDDPFDFGRIAATNALSDVYAMGGKPILAL-ALVGMPINVLPH----ETIAAVLRGGEA

Burmal IVDDPFDFGRIAATNALSDVYAMGGKPILAL-ALVGMPINVLPH----ETIAAVLRGGEA

Sinmel IVDDPRDFGRIAATNAISDVYAMGGKPILAL-AIVGMPINKLDS----TTIAKILEGGAS

. *

Metjan FCRENKTTIVGGHTILNPWPLIGGAVTGVGREEEVLTKAGVKVGDVLILTKPLGTQTAMA

Metmar FCRENDTTIIGGHTILNPWPLIGGSITGVGKEEDILTKAGCKNGDVLILTKPLGNQSAMA

Metkan QCREVDALIVGGHTIMNPWPILGGCVTGFA--ERYVTVGGAEPGDVLYLTKPLGTQPAMA

Homsap AAEEAGTSVTGGQTVLNPWIVLGGVATTVCQPNEFIMPDNAVPGDVLVLTKPLGTQVAVA

Dromel SALEAGTTVTGGQSVVNPWCTIGGVASTICQPNEYIVPDNAVVGDVLVLTKPLGTQVAVN

Caeele AADEAGTKIRGGQTVRCPWLLLGGVATSVAHESEIIKVDQAVPGDVLILTKPIGGQVAVN

Aquaeo KLREAKTVLLGGHTIDDKEPKFGLSVAGICPEGKYITQSGAQVGQLLILTKPIGTGILIK

Cloper KVLEAGAVIVGGHTVQDDEPKYGLSVTGIVHPDKVLKNYGSETGDILILTKPIGLGIINT

Camjej KVKECNALVVGGHTIESTELFFGLSVTGKVHPSKFIANNTSKIGDCIILTKPLGTGILST

Helhep KITESNALLLGGHTIKDKEQKYGLAVNGIAHKNRLWRNHTGYIGDMLVLTKPLGSGILTT

Wolsuc KIIESQGILIGGHTIDDREQKYGLSVTGFVHPQKIWRNHTTQEGDALILTKPIGMGILTT

Entfae KLKEAGALLAGGHSIYDHEPKYGLAVTGEVHPENLWHNNTPQVGDVLILTKALGVGIIQA

Porgin TIDESGAFTMGGHTIDDPIPKYGLAVTGIVHPEHLVTNAGVRAGQCLVLTKPLGIGVAMA

Mycavi VCGLAGCHLAGGHSVDDPEPKYGLAVTGIADPNRLLRNDSGKPGLPLSLTKPLGIGVLNN

Symthe KVAEAGAVLIGGHSIDGPEPTYGLAVTGLVHPDRVGAKAGVRPGDRLILTKPIGVGVITT

Bdebac KIAEADANFVGGHSIDDDTLKFGLSVTGFVNPQQVWTNAGAKVGDHLILTKPLGTGTLTA

Niteur KIAEAGANFVGGHSIDDDTLKFGLSVTGFVNPRQVWTNAGARPGDHLILTKALGTGTMTA

Theten KVKEAGALIAGGHTIEDEEPKYGLSVTGIVHPEKVIKNSTAKPGDVLILTKPLGIGVINT

Geosul ALREAGACLVGGHTVEDDELKYGLAVTGLISPSRVVRNATARAGDRLVLTKPLGTGIVST

Treden KVYEAGAIVCGGHTIYDDSPKYGLAVNGFVHPKKILENSTAKEGDVLILTKPIGTGILLT

Despsy MLREANTVLLGGHSVEDEELKYGLSVTGFVHPDKILANQGLRTGDVLILTKAIGTGILNT

Desvul KVREAGAVLVGGHSIEDESIKYGLSVTGIIDPDCYATNTGLRPGDVLLLTKPLGSGVLAT

Phopro2 KVREAGAVLVGGHTVEDDEPKFGLSVMGMIHPSKVQTKAAVESGDILVLTKPLGTGVIST

Haeinf ACHQAGIALAGGHSIDSPEPIFGLAVTGVIDTEKVKRNASAKSGCKLYMTKPLGIGILTT

Pasmul ACRQAGITLAGGHSIDAPEPIFGLAVTGIINTDKVKKNASATAGCKLFLTKPLGIGVLTT

Mansuc ACREAGIALAGGHSIDAPEPIFGLAVTGIVPTEKVKRNASAEAGSKLYLTKPLGIGILTT

Yerpes VCQQAGISLAGGHSIDAPEPILGLAVTGIVSTEQVKKNSAAKPGCKLFLTKPLGIGILTT

Yerpse VCQQAGISLAGGHSIDAPEPIFGLAVTGIVSTEQVKKNSAAKPGCKLFLTKPLGIGILTT

Pholum ACKEAGIVLAGGHSIDAPEPIFGLAVTGIVNIDRVKQNSAAKVGSQLFLTKPLGIGVLTT

Saltyp ACRQAGIALAGGHSIDAPEPIFGLAVTGVVPTERVKKNSTAQAGCKLFLTKPLGIGVLTT

Shifle ACRQAGIALAGGHSIDAPEPIFGLAVTGIVPTERVKKNSTAQAGCKLFLTKPLGIGVLTT

Esccol ACRQAGIALAGGHSIDAPEPIFGLAVTGIVPTERVKKNSTAQAGCKLFLTKPLGIGVLTT

Phopro1 VCREAGISLAGGHSIDAPEPIFGLAVTGIVDTDRVKRNNRAENGCKLYLTKPLGIGVLTT

Pseput VCAEAGIPLAGGHSIDAPEPIFGLAVTGVVSKRHLKRNDTASAGCQLYLTKPLGIGILTT

Psesyr VCDAAGIPLAGGHSIDAPEPIFGLAVTGVVQKKHMKRNDTAQAGCVLYLTKPLGIGILTT

Pseaer VCEEAGIPLAGGHSIDAPEPIFGLAVTGVVEKRFMKRNDTAEAGCRLYLTKPLGIGILTT

Borbro ACDEAGIALAGGHSIDAPEPIFGLAVTGVVDKARLKRNDTATAGCRLYLTKPLGIGILTT

Sheone ACMEAGIMLAGGHSIDAPEPIFGLAVTGQIALTDLKQNDTAKAGDRLYLTKPIGIGILTT

Haeduc ACQQAGIVLAGGHSIHSMEPIFGLAVTGMAAIEHIKRNASATAGCELFLTKPLGIGILTT

Burpse VCADAGIPVAGGHSIDSVEPIYGLAALGVVHPARVKRNAAARAGDVLVLGKPLGVGVLSA

Burmal VCADAGIPVAGGHSIDSVEPIYGLAALGVVHPARVKRNAAARAGDVLVLGKPLGVGVLST

Sinmel ICAEAGIPVAGGHSIDSVEPIYGLAVIGLCHPSEVRRNGGVKAGDALILTKGIGVGIYSA

**::: * * : : * :*

Metjan LSRIPE----EFKDLISITEEERDYIINKAIEIMTTSNRYALKALRKAEERVGDKIANAL

Metmar LSRVTE----EFEDLIDIPKEEQKYIFEKTIELMTTSNRIALLHLRELENELGEKIANAM

Metkan ALRLPE----DVRKQFLTDSELEEAV-DLAVEVMTEPLKDAAEAALE-------VGVHAM

Homsap VHQWLDIPEKWNKIKLVVTQEDVELAYQEAMMNMARLNRTAAGLMHT-------FNAHAA

Dromel AHQWIDQPERWNRIKLVVSEEDVRKAYHRAMNSMARLNRVAARLMHK-------YNAHGA

Caeele SYEWIKKKNGKIEELNLEIP-KIEKAFKQVCEQMSRLNRNAAKLLHK-------YDAHSS

Aquaeo GLKEGI----------LKEE----DI-NEAIENMLALNDKARNLMLS-------LDATAC

Cloper AIKAKI----------ASKE----AY-EKAVKVMAYLNKYAGEIITD-------YNITSC

Camjej ALKAQM----------LNQK----HL-DIMLKNMIELNYKASQIALK-------FHPSAM

Helhep AIKAQM----------FSQT----T---EVTQSMAMLNLYAARIAQN-------YEIHAC

Wolsuc SIKADM----------LSPQ----ST-HEAGELMATLNLYAAQIASE-------FEIHAC

Entfae AVRGQL----------AKQT----EQ-KIAQDSMERLNKYAAANARS-------YAVHAC

Porgin AHRLGL----------IGSE----VY-EAAIGQMCLLNRAGAELMQK-------YGIRGA

Mycavi RHKAT-------------GE----RF-EEAIAVMTTLNAEAAGAALA-------AGVECA

Symthe AIKRGL----------ARPA----DV-DEVTQVMAKLNNVV-DVLAP-------FDIRGM

Bdebac GLKRQE----------VQEA----DI-MEALQSMATVNNAVDYMTPV-----LKNEVHAA

Niteur AVKRQQ----------LREE----DI-IEALDSMTAINNAIDCMSPD-----LQASIHAA

Theten AIKGEM----------CPSE----TY-LLAVEVMKYLNKEASEIMKE-------VGVNAC

Geosul AIKADM----------APAA----LT-AEAVRWMTMLNAEAAGLMLE-------CGASAC

Treden ASKADM----------SPPE----EL-DRCYKIMAFLNAKARNIMVK-------YKINAC

Despsy AIKARL----------ASKE----TI-EELTELMVTLNKKPAEIMRE-------FNISAC

Desvul AVKAGW----------DGFE----AHEQELGRWGAMLNRAGGRVIRE-------LGLAAA

Phopro2 AAKRGK----------ASQE----SI-QTSTDSMKKLNRNAAQIFVK-------YPIKAC

Haeinf AEKKGK----------LKPE----HQ-GLATAAMCQMNSIGSQFSQV-------DGVTAM

Pasmul AEKKGK----------LKAE----HQ-GLATEVMCQMNTIGAKFAEI-------AGISAM

Mansuc AEKRGK----------LKPE----HK-GLATEVMCQMNLIGSQFSQL-------ESVTAM

Yerpes AEKKSK----------LRPE----HR-GLATETMCQLNKPGADFAHI-------PGVTAM

Yerpse AEKKSK----------LRPE----HR-GLATETMCQLNKPGADFAHI-------PGVTAM

Pholum AEKKGL----------LLPE----HQ-GIAIETMCRLNKLGMDFAEV-------AGITAM

Saltyp AEKKSL----------LKPE----HQ-GLATEVMCRMNVAGAAFANI-------DGVKAM

Shifle AEKKSL----------LKPE----HQ-GLATEVMCRMNIAGASFANI-------EGVKAM

Esccol AEKKSL----------LKPE----HQ-GLATEVMCRMNIAGASFANI-------EGVKAM

Phopro1 AEKQSK----------LADE----HK-GLARDWMCKLNIPGQDFANV-------EGVKAM

Pseput AEKKAK----------LRVQ----DQ-GLARDWMCTLNTPGSRFGKL-------DGVKAM

Psesyr AEKKAR----------LREE----DM-GLARDWMCTLNTPGSRFGKL-------AGVAAM

Pseaer AEKKAR----------LRAE----DV-GVARDWMCTLNRPGARFGRL-------AGVKAM

Borbro AEKKSR----------LRPE----DV-NLARDWMCTLNKPGSRFGAL-------AGVKAM

Sheone AQKQKK----------LKDE----DS-HIAVNAMCQLNSIGAQIAKI-------NGVNAL

Haeduc AEKRGL----------LTLP----HQ-HLVRDLMCQLNTIGTLLAPL-------PEMTAM

Burpse ALKKDR----------LDAQ----GY-AQMIATTTKLNRPGTALAAL-------PGVHAL

Burmal ALKKDR----------LDAQ----GY-AQMIATTTKLNRPGTALAAL-------PGVHAL

Sinmel AIKKNA----------LPPG----CY-EEMIGSTTLLNRIGADLAAD-------PDVHAL

.

Metjan TDITGFGILGHSNEMAKN----SNVLIEINL--LPCIKRTPELSR--LF--G--------

Metmar TDVTGFGILGHSQEMAEQ----SDVE--IEISCLPVIKGTPELAS--LF--G---HALCS

Metkan TDVTGFGLKGHAGEMAEA----SGVR--VVIERLPVIPGTTELSR--ALGYGLER-----

Homsap TDITGFGILGHAQNLAKQ----QRNEVSFVIHNLPVLAKMAA-VSKAC---G---NM--F

Dromel TDITGFGLLGHAQTLAAH----QKKDVSFVIHNLPVIAKMAA-VAKAC---G---NMFQL

Caeele TDVTGFGLLGHAENLARV----QKQPMEFIIEKLPIIEYMDEIADKMIAKGG--EGFKLY

Aquaeo TDVTGFGLLGHAWNICKN----SNIGARIFFEKVPYYQLSENLVKKKIYPKGAIENLNFV

Cloper TDITGFSLIGHAYEMAEP----SKKTFRIFKDAIPFIKEAKEYASMGLIPAGCYENKRYL

Camjej SDVTGFGLLGHLKEMLN-----KNISFEIFESELPFLDGVKEYFNMGLIPAGAYKNLEFI

Helhep TDITGFGLIGHAFEMCGGIKNQNEKSILFYTKQIPLFDKTESFSQMGIVPGGSYENKKAL

Wolsuc TDVTGFGLLGHALEMCGN------RSIRLFASQTPYLSEALEMAKMGIIPAGSYANKSYL

Entfae TDVTGFGLLIHAKEMADN-----QVTLLIDTEALPTITGALAYAAECLVTAAGQRNRQTI

Porgin TDITGFGLLGHAKGLAEA----SDVCLHIDSRSVPVLPECLSLLRDGCIPGAAFRNLRFV

Mycavi TDVTGFGLLGHLHKLARA----SGVTAVLDAAAVPYLDGAREALAAGYLSGGTRRNLDWV

Symthe TDITGFGLLGHASEMART----SGVGLRLSAGAVPVLPSTYDYAEQGAFPGGSKANFRFL

Bdebac TDITGFGLSGHGMQLANA----SQVSLRISFSKIPRFAKALAFLEKGFLTKAHRSNAEYT

Niteur TDITGFGFSGHAMQLANA----SDVTLCIETGNLPRFDKTFHCLENGCLTKAHRTNAEYT

Theten TDITGFGLLGHAYEMAFS----SGVTIEFDKDSIPLIEGARELAQMGLIPGGCYRNKKYL

Geosul TDVTGFGLVGHACEVARG----AGVTLRLHLEQVPVLDGVMGLVADGLVPAGCYRNRDHY

Treden TDITGFGLLGHLYEMGKG----SGMSIEVDYKSVPIYKSVIESAEMGMMPAGVYSNRNFV

Despsy TDITGFGLLGHIAEMIDG----SDKSITIQSQNVPILNQAHHFASMGLVPIGAHNNRAFR

Desvul TDVTGFGLGGHLLEMANA----SNMSVHVDVSTLPLMPAVLDLVATGLLPAGSHANRHFC

Phopro2 TDITGYSLLGHALEMAEK----SDVCMHFIADQVPFLLGAEDYAAQGIFPGGANRNLEAY

Haeinf TDVTGFGLLGHLIEICEG----SNLSAVVFSDKIKTLDGVKDYIAQGCVPGGTGRNFDSY

Pasmul TDVTGFGLLGHLSELCEG----SGVRAEVYFDKIKTLDGVQRYIEKGCIPGGTERNFESY

Mansuc TDVTGFGLLGHLAEICEG----SNLVADVHFNKIKMLDGVPYYIEQGCLAGGVTRNYESY

Yerpes TDVTGFGLLGHLSEICQG----SGVQAILHYSAIPRLPAVEDYIAEGCVPGGTGRNFDSY

Yerpse TDVTGFGLLGHLSEICQG----SGVQAILHYSAIPRLPAVEDYIAEGCVPGGTGRNFDSY

Pholum TDVTGFGLLGHLSEICAG----SGVQATLHFAKVPKLPEVESYIAKGCVPGGTGRNFDSY

Saltyp TDVTGFGLLGHLSEMCQG----AGVQAMLCYQDIPKLPGVEEYIALGAVPGGTERNFASY

Shifle TDVTGFGLLGHLSEMCQG----AGVQARVDYEAIPKLPGVEEYIKLGAVPGGTERNFASY

Esccol TDVTGFGLLGHLSEMCQG----AGVQARVDYEAIPKLPGVEEYIKLGAVPGGTERNFASY

Phopro1 TDVTGFGLMGHLSEICEG----SQLKARVDFDSVPYLPGVFDYIAQGCVPGGTTRNFDSY

Pseput TDVTGFGLLGHLVELAEG----SGLTAHLNYAAVPRLPSVDHYLAEGCIPGGTLRNYDSY

Psesyr TDVTGFGLLGHLVEMADG----AGLTARLDYAAVPRLPGVEHYIAEGCVPGGTLRNFESY

Pseaer TDVTGFGLLGHLVEMADG----SKLTARVEYAAVPRLASAEYYLEQGCVPGGTLRNFDSY

Borbro TDVTGFGLLGHLVEMADG----SGVSARIEYAKVPRLPGVEHYLAEGCVPGGTGRNFDSY

Sheone TDVTGFGLAGHLLEMCQG----AKLTAKLKLDAVPLLPRALDYLAQGCVPGGTHRNYDSY

Haeduc TDITGFGLLGHLSEICQA----SNVRAEINSHAVKVIDGVEEYVEQGMIPGGTQRNFESY

Burpse TDVTGFGLLGHTLELARG----AGLTARVRYGALPWLAGVEALVADGVLTGASGRNWAAY

Burmal TDVTGFGLLGHTLELARG----AGLTARVRYGALPWLAGVEALVADGVLTGASGRNWAAY

Sinmel TDVTGFGVLGHGLEMARA----SQLSLTLRLSAIPFLSQAYMLAEQGFITGASGRNWASY

:*:**:.. * : . .

Metjan --------HALLDG------YGAETAGGLLISAKEEYKDN--LIDELEKA----KC-YAF

Metmar GK-------------------GAETAGGLLISTKPEYKDK--LIQKFKEN----NV-YAF

Metkan GE-------------------SAETAGGLLVAVPEEHAED--LEDAFERR-----DVWYR

Homsap G-----LMHGTC----------PETSGGLLICLPREQAAR--FCAEIKSP-KYGEGHQAW

Dromel -----------LQG------HSAETSGGLLICLPREQAAA--YCKDIEKQ---EGY-QAW

Caeele QG------------------TSAETSGGLLIAMSEENAKK--YIAELSSL----DNAPAW

Aquaeo KN--YL--KSNLDNWKLILLSDPVTSGGLLFTINKEKLEK--IDETAKEL----EV-NYW

Cloper EG---KYLLNNVESWMEDILFDPQTSGGLLISCKEKDYID--ILTRLEKL----EV-ESA

Camjej KE--LT---PDLNE-EKLLLCDPQTSGGLLISISEKDSLE--CLKKLEDE---N--IQAK

Helhep QS--QVQIQCTLED--DIFYYDAQTSGGLLFALPFNQAKL--FVDELHKA---GII-HAN

Wolsuc EP--LTSIEASLSAEEAMLFYDAQTSGGLLFSLSAKEAPK--LLEALRQG---GI-SHAS

Entfae GT--TIDYS-KISMEMQELLFDPQTSGGLLISVAADEAAA--CLAAIQKE---D-P-VAK

Porgin GD--ML--RADCPTEYKMLLADAQTSGGLLMAVDADRAED--LVADLHRT---GLHPFAA

Mycavi AP--HVDLS-GVGEDEALLLADAQTSGGLLIAGE---------------------IPGAP

Symthe TGEGHVEFAESVPELVRMILCDAVTSGGLLIAVEAARAAD--LASALTAH---GTL-AAA

Bdebac KE--AISV-AGLESLQQHLLHDPQTSGGLLLSVSREVSAD--MVQALRAK----FK-SAE

Niteur TP--HID-DSTLDALHKLLIHDPQTSGGLLLSVAPETSQT--MLQALHTR----FP-SAV

Theten KG--KVCIKVE-EDEVIDLMFDPQTSGGLLISVSEEKAEE--LYRRLNKK---LKF-GAF

Geosul AP--FVGAPRS-DDDRLLPLFDPQTSGGLLLSLSPSSAGR--FLAAAGDR---G--LFAL

Treden GD--NIVFE-NVPLAYQDLMFDPQTSGGLLISVDKEDAAA--LYEELSQALENTPCGKPA

Despsy KE--MMQIPKAFDPVLRDILFDPQTSGGLLIGCAEEDGPS--LVRRLQDA---GV-KDAQ

Desvul SG--NVSVHPEVDSLLVDIVFDAQTSGGLILAVPPHLVDD--ACSILRAE---D--APFW

Phopro2 KD--DIEFAPELDESWQQKLCCPETSGGLLATVPKDCLES--LLLEFSNT---G--ESCW

Haeinf GH--KV---GILTEEQKAILCDPQTSGGLLVAVELNSVQT--VIDIAKDA---G-ID-LY

Pasmul GH--KI---GAMSDLQKAILCDPQTSGGLLIAVDADSEQQ--VLEIAADD---S-IE-LF

Mansuc GI--KI---GAITEFQKAVLCDPQTSGGLLVAVKPEGETQ--LLELAAQA---G-IE-LI

Yerpes GH--LI---GNMSDLQKQLLCDPQTSGGLLLAVLPDAEAD--VQAIAAQH---G-MT-LS

Yerpse GH--LI---GNMSDLQRQLLCDPQTSGGLLLAVLPDAEAD--VQAIAAQH---G-MT-LS

Pholum GH--LI---GEMTELQRKLLCDPQTSGGLLLAVLPEAMDE--VKAIARCH---G-IE-LT

Saltyp GH--LM---GDMSREVRSLLCDPQTSGGLLLAVTPDAEDD--VKATAAEF---G-IE-LT

Shifle GH--LM---GEMPREVRDLLCDPQTSGGLLLAVMPEAENE--VKTTAAEF---G-IE-LT

Esccol GH--LM---GEMPREVRDLLCDPQTSGGLLLAVMPEAENE--VKATAAEF---G-IE-LT

Phopro1 GQ--KL---GAMTEQQKALLCDPQTSGGLLIAVTPDAETEQQLQAIAARH---N-IE-LQ

Pseput GH--KI---SALSDDQKHLLCDPQTSGGLLVAVAPEGEAE--FLAVAAEL---G-LK-LS

Psesyr GD--KI---AALSDAQRDLLCDPQTSGGLLVAVSPEGEAE--FLAVAAEL---G-LQ-LN

Pseaer GE--RI---APLPEVQKLLLCDPQTSGGLLVAVAPEGEAE--FLAVAAEL---G-LQ-LA

Borbro GA--RI---APLPQDRVDLLCDPQTSGGLLVAVEPAGEAE--FLAAAGEL---G-LR-LE

Sheone GE--HL---PELTDHQKAILCDPQTSGGLLVAVSSEAEAE--LVALLNAN---Q-IE-PI

Haeduc AN--LV---SPLSDRQKAILCDQQTSGGLLIAVEPQAVEK--IQQIAQQI---G-SM-LF

Burpse GH--DVRLGDGLPAVAQALLTDPQTSGGLLVACAPEAVDD--VLACFRDD---G-FERAA

Burmal GH--DVRLGDGLPAVAQALLTDPQTSGGLLVACAPEAVDD--VLACFRDD---G-FERAA

Sinmel GA--DVVLPDDLPDWQRLLLADPQTSGGLLVACAPEKAGA--LVEKVRLA---G-YPLAG

*:***:

Metjan EVGRVVKKGEGKAVLSKDVKVIEI----------------

Metmar EVGKIVNNGVGIAKLSENVEILEI----------------

Metkan RIGRVEEGSGVEVRGDVEEVEDYP----------------

Homsap IIGIVEKGNRTARIIDKPRIIEVAPQVATQNVNPTPGATS

Dromel IIGIVEKGNKTARIIDKPRVIEVPAKD-------------

Caeele IIGKVTAKTTDSSIARILPDAVRISVPSHI----------

Aquaeo IIGETIAENVLEVL--------------------------

Cloper VIGRVEDF-NDAYIVVE-----------------------

Camjej IIAKVVNK-QENDIIIS-----------------------

Helhep VIGEIIPK-TETSIVLG-----------------------

Wolsuc LVGEVLPRGEKPLYIG------------------------

Entfae IIGEVLPK-EQQAVVLL-----------------------

Porgin IIGYATDAEDAAKLIVT-----------------------

Mycavi VIGELVPRGEHTIVVR------------------------

Symthe VIGEAVAE-HPGKIMVID----------------------

Bdebac IIGEVLPR-QDKAVIFEP----------------------

Niteur IVGTVHPHQDKAVQFA------------------------

Theten IVGRVKEK-QEYDIYVR-----------------------

Geosul EVGEVLPAGECAVDIV------------------------

Treden IIGLVTKRDEKILRVS------------------------

Despsy IIGFVTENRANIIQIS------------------------

Desvul RIGHVEEMGEGVPRLVLQP---------------------

Phopro2 VVGYAESGSGIKVS--------------------------

Haeinf EVGKLKPK-SESDIVVEVK---------------------

Pasmul EVGRLFAKEHDSPILITVL---------------------

Mansuc EVGELRRRVDNSDPVIIRILD-------------------

Yerpes PIGELTSADSRRALIEIVV---------------------

Yerpse PIGELTSA-DSRRALIEIVV--------------------

Pholum AIGELSEQ-QSGRVLIEVNE--------------------

Saltyp AIGELVEARGGRAMVEIR----------------------

Shifle AIGELVPARGGRAMVEIR----------------------

Esccol AIGELVPARGGRAMVEIR----------------------

Phopro1 AIGEMMPLDGDTLIEIC-----------------------

Pseput PIGKLVERQSHAVEVI------------------------

Psesyr PIGTLLERQTHAVEVF------------------------

Pseaer PIGELVER-QSLAVQVL-----------------------

Borbro PIGELVAQRPYAVEVL------------------------

Sheone CIGSLEAPTTATNVVLC-----------------------

Haeduc HIGKLFDRAPDKALIEVN----------------------

Burpse AIGEMVDGAARVDVS-------------------------

Burmal AIGEMVDGAARVDVS-------------------------

Sinmel IVGTAAAGAAQIKVIS------------------------

:.

**YBBB**

CLUSTAL FORMAT for T-COFFEE Version_1.37, CPU=0.00 sec, SCORE=51, Nseq=19, Len=429

Camjej -----------------MLSEVEFVEFQKENFSLLIDARSPREFLHSHLIGALNFYALND

Wolsuc ---------------VLEELTLERFLSHRSAFSLVIDVRSPHEYLESHLPGALNLPVLSD

Burpse ------------------MNLQPASLAQIDDFDEIVDVRTPLEYAEDHIPGALNAPVLSN

Cloper ----------------MFGLISYEEIYGHEDEYIFIDVRSQKEAFEEPMIGSVNIPVLLN

Despsy MKKEKEGQAIDPETMLAEALHSVAFTDPSYGDYLVVDVRTHAEFIENSLPGAINIPLFDE

Desvul ---------------MPHIIDASTFLALRDTGLPVLDARSPSEHARGAIPGALNLPVLDD

Porgin ------------------MLIDTDFFLSNRSDSLVLDVRSPEEYRRGHIPGAESFPLFSD

Geosul ------------------VIKTTPFHESLLDTHLVVDVRTPLEYEEDHLPGAINVPLLTN

Bdebac -----------------------------------MDVRAPVEFSQGSIPGAVNLPVMND

Niteur --------------LRNPDIGIDDLTALFIADTPLIDVRAPVEFTQGSLPGAVNLPILND

Borbro -----------------MRADTRDFRALFLGDAPLLDTRAPVEFAKGAFPGAVNLPLMSD

Pseaer -----------------MRDNTQHYRELFLDDIPLMDVRAPVEYHKGAFPNTVNRPLMND

Pseput -----------------MRPDCTDFRQLFLDDVPMMDMRAPVEFAKGAFPGVVNLPLMND

Psesyr -----------------MADNSSDYRALFLNDVPMMDARAPVEFSKGAFPGVINLPLMND

Phopro --------------MLMPRQNCEVFRQLFVNDTPLMDMRAPIEFAQGAFPTSINHPLMED

Sheone --------------MTTTLIPAQQYRDIFVAGKPLIDLRAPIEFNRGAFPSSVNLPLMVD

Esccol ---------------MQERHTEQDYRALLIADTPIIDVRAPIEFEHGAMPAAINLPLMNN

Shifle ---------------MQERHTEQDYRALLIADTPIIDVRAPIEFEQGAMPAAINLPLMDN

Saltyp ---------------MQDRQKAQDYRALLLADTPLIDVRAPIEFEQGAMPGAINLPLMMD

:* *: * . : . : :

Camjej EEYQEIGTIYKKNQALAK-AMGASYICQNTTKHILEITQNF-RI-GEKVGIYCSRGGLRS

Wolsuc LEHERVGTLYKESPFEARI-LGASLICANIASALSWL-KDSLHP-SQKVLIYCARGGQRS

Burpse EERVIVGTMYKQVSPFEATRVGAAMVARNIAAHLDTTFAD-RPR-NWRPLIYCWRGGKRS

Cloper EERDVVGTLYVRESAEAAKEQGIEFISRRLPEIFKQVQELYRNKHGKKLVIFCARGGMRS

Despsy MERSVIGTLYKQVGRQEAVQAGFEIVHPKLSAI-VESFEPY---RQRKLLISCARGGMRS

Desvul AQREAVGTLYARSGREAAVHLALQLVGPHLSAMLARAQHL-CEN-RHEVLVHCWRGGMRS

Porgin AERAEVGTLYVRHSRQAAVERGLEFVGPRMAEMVREARS--LAA-GRDIILYCWRGGMRS

Geosul EERVEIGTIYKQTGPLEARRRGLHLTAHRFPAMVGEIAR--AAA-GRPILVYCWRGGLRS

Bdebac DERAQVGTVYKNQGNEAAVKLGHELVSGLVKEQRVAAWKSFVQA-HPEAVFYCFRGGQRS

Niteur EERALVGTTYKQQGSEAAIKLGYEMVSGSVKQNRLQQWLDFIHQ-HPRAILYCFRGGKRS

Borbro AERHKVGLCYKQQGQDAAIALGHRLVSGAVKAERVAAWAAFAQA-HPEGYLYCFRGGLRS

Pseaer IERQKVGTSYKQHGQQAAIALGHELVCGALKAERLAAWKAFAEA-NPNGYLYCFRGGLRS

Pseput QERQKVGTCYKQQGQAAAIALGHQLVSGATKQARLEAWAAFAKA-HPDGYLYCFRGGLRS

Psesyr IERQKVGTCYKQHGQDAAIQLGHQLVCGQVKDERVEAWVEFARA-NPNGYLYCFRGGLRS

Phopro EERKAVGTCYKAHGQDAAIALGHQLVNGDIKAARLAQWKAFAEE-NPNGYLYCFRGGLRS

Sheone KEREKVGTCYKQQGQQAAIALGHSLVHGAVKQQRIDAWLGFLAA-QPEAYLYCFRGGLRS

Esccol DERAAVGTCYKQQGSDAALALGHKLVAGEIRQQRMDAWRAACLQ-NPQGILCCARGGQRS

Shifle DERASVGTCYKQQGSDAALALGHKLVAGEIRQQRMYAWRAACLQ-NPQGILCCARGGQRS

Saltyp DERAAVGTCYKRQGADAALALGHRLVCGDIRQQRLEAWKAAYQR-FPNGYLCCARGGQRS

: :* * . . * *** **

Camjej KSIAVILS-ELGFRVVRLKGGFKAYRTFVTHYFEN-EINFDFFALCGNTGCGKTELLEQL

Wolsuc LSLALILS-SIGYQIARLIGGYKGYRAHVAASWQV-PRKESFLTLVGPTGGGKSELIQSL

Burpse GSMTVWFN-MIGWRARQLDGGYKTYRRSIVDALGELPAGFRYVVLAGHTGSGKTRLLHAL

Cloper GSLHSLLN-SLGINCYKLEGGYKAYRSFMINKIEEFSKEIDFIVLYGNTGVGKTEMLYKL

Despsy RAVVNLLA-GQGFNIAQLEGGYKAYRHTVLDRVKNFAP--EMIVLHGMTGVGKTRIIEKL

Desvul GSLAWLLE-SAGYTVHLLEGGYKAYRAHVRAQLAR---PARVIVLGGMTGTGKTDILHAM

Porgin ESVAWLLS-VAGLRIRRLRGGYKAYRRHFPDILKLY--PWRFIVLGGYTGSGKTPILNEL

Geosul KTVVSILD-LAGFDAVQLQGGHKAFRHLVLSSFDPFTPPAPLVVLHGMTGVGKTTFLLQL

Bdebac RISQAWLK-EAGVERPLLVGGFKAARNYLIKQIEEFSVRQELISVSGPTGSGKTHFLRGL

Niteur QITQQWLR-DTGIDSPLITGGYKRARQFLISTIDRFSEHRKLLVITGPTGSGKTRLIHDI

Borbro QISQAWLREEAGIAYPRVIGGYKAMRGFLLETIEQAIAECGFVVLGGMTGTGKTDVLRQL

Pseaer QIVQQWLKQDAGIDYPRVIGGYKALRNFLFETTRAAVDECDFVLVGGLTGCGKTEVIAAL

Pseput QIVQGWLRDEAGIQYPRVKGGYKAMRTFLLETTQQAVEQCDFVLVGGLTGTGKTDVLHQL

Psesyr QTVQRWLK-DAGVDYPRILGGYKAMRTFLLDTLHEAVTECDLVVLGGMTGTGKTEVLTQL

Phopro RITQQWLK-EAGIDYPMVVGGYKALRRFLIETIDTV-AQQPMTIVGGNTGSGKTIMVNEL

Sheone QLTQQWLK-EAGATVPYVQGGYKGMRQYLIGVIETTPSQQPLLSLSGMTGSGKTDFLIQR

Esccol HIVQSWLH-AAGIDYPLVEGGYKALRQTAIQATIEL-AQKPIVLIGGCTGSGKTLLVQQQ

Shifle HIVQRWLH-EAGIDYPLVEGGYKALRQTAIQATIEL-AQKPIVLIGGCTGSGKTLLVQQQ

Saltyp HIVQRWLQ-ETGIDCPLIEGGYKALRQTAIQATWQL-AQKPILLIGGCTGSGKTQLVRQQ

: * : **.* * : * ** **: .:

Camjej PQA----INLEKMANHLGSSFGDILG-KQPTQKAFEAELFHNIQNLE------NFAFIES

Wolsuc SWS----LDIEGLARHKGSSFGAIEG-EQPSTKMFQNLLFERLLGLREE----PLVAVEG

Burpse ARAGAQTLDLEALAAHRGSLLGALPSGEQPSQKAFDTALVSALRGFEHD----RPVFIEA

Cloper EEKGYDVLDLEGCANHRGSILGGVGLGECNTQKRFDALVYDKLR-HRNS----NIVFIEG

Despsy SPS----IDLELMANHRSSLFGALDR-EASNQKGFEAAFYQKI-----EEGGQEPFFVEG

Desvul ADLGCQVVDLEGLANHRGSAFGSIGLGKQPTNEQFEAALYEVWSRLDHG----RPIWMED

Porgin TQMGEQVIDLEGLAHHKGSAFGALGQEQQPTTEHFMNLLHDALSACDPN----RPIWVES

Geosul ARAGHAVVDLEGLARHRGSAFGELGLRQDISQKRFETLLWDAFRRLPAG----CPILLEG

Bdebac KG--CPVIDLEALARHRGSAFGNWEV-PQPTQVDYENNLAREILLLEDKIHGKIKPLVED

Niteur SNS-HPVLDIEALARHRGSAFGGMSV-PQPSQIDFENHLAVNLLKLEQNNLSE-PVIVED

Borbro DHG----LDLEAHAHHRGSSFGKHAT-GQPAQIDFDNRLAIDILKKRAA-GC-RQFVVED

Pseaer DNS----LDLEGHANHRGSSFGRRAT-PQPAQIDFENRLAIDILKKRHR-GV-GQFVLED

Pseput DNV----LDLEGHANHRGSSFGKRAT-AQPAQIDFENQLAIDVLKKRAR-GI-GQFVLED

Psesyr PNG----LDLEGVANHRGSSFGKRAT-GQPAQIDFENRLAINLLKKRAA-GI-EQFVVED

Phopro ANG----IDLEGAANHRGSSFGRYVT-AQRTQIDFENVLAVEMLKKQAQ-GC-THFVFED

Sheone KEA----VDLEGIANHRGSSFGKNID-PQPTQINFENQLAIALLRHQQD-NH-SCLLLED

Esccol PNG----VDLEGLARHRGSAFGRTLQ-PQLSQASFENLLAAEMLKTDARQNL-RLWVLED

Shifle PNG----VDLEGLARHRGSAFGRTLQ-PQLSQASFENLLAAEMLKTDARQDL-RLWVLED

Saltyp PNG----VDLEGLARHRGSSFGRTLK-PQLSQASFENKLAVELLKINARQTL-KRWVLED

:::* * * .* :* : . .*

Camjej ESRKIGDIILPLKFYEKMQKAFKIYCFCSLENRVRRIQKIYQEKMTP-------------

Wolsuc ESKRLGNLILPSPLYERYKSAPKVFIDSPLEARIDRTMQEYASISPAFF-----------

Burpse ESRRIGAITLPLALLAGMHAADCVKVETARDERVELLLQDYGHLFDQ-------------

Cloper ESKRIGRILIPDPLFEKMYEGAKIKISADLEIRAERLVKEYTA-----------------

Despsy ESRKIGQVFMPDRFAVAMKQARMVFVTASMETRVQRIIEDYPMDDPQ-------------

Desvul ESSRIGTVAMCDAFFTHIEHGRLVTVELPLEARVARLVAEYAAT----------------

Porgin ESKTIGRVFLPDDFYKVMRQAPLIELSVPRPVRIAHIAKEYGVY----------------

Geosul ESRRIGRLTLPGNLYDVMRGSVKLWCEASVATRVARLTEEYGR-----------------

Bdebac ESRLIGRISQPATFFIRLRSSPVIWIDEPLPVRVDNVFDDYILNSSIGKS------LEAA

Niteur ESRHTGKVYLPDSFFHHLRNSEIIWVDEPLATRVDNIFEDYILTTPIGQAQRIRQAIPPL

Borbro ESQAIGSCSLPFGLYQGMQRYPVVWLEDTQPARVQRILRDYVIDLCGEFT----------

Pseaer EGRIVGSCSLPLELYQGMQGYPLVWLEDAFEQRVERILRDYVIDLRSEFE----------

Pseput EGRIVGSCTVPLELYQGMQHYPLVWLEDSFTNRVERILRDYVVNLSAEFK----------

Psesyr ESRLVGSCNVPLELHQAMQGCPVVWLEDSFEHRVERILADYVVNLCAEFI----------

Phopro EGRAIGSASVPLSINAAMGNADVAIVDDPLDVRIDRLIDDYVVRMQRDYI----------

Sheone ESFLIGRSALPQSFYNAMQAADILVLEEADDIRLNRLLDEYVHKMHQGFV----------

Esccol ESRMIGSNHLPECLRERMTQAAIAVVEDPFEIRLERLNEEYFLRMHHDFT----------

Shifle ESRMIGSNHLPECLRERMTQAAIAVVEDPFEIRLERLNEEYFLRMHHDFT----------

Saltyp EGRTIGANHLPECLRERMAQAPIAVVEDPFALRLERLREEYFIRMHHDFT----------

*. * : * *

Camjej -------------------LKFQQCVQKISPYISLNLRQDLLQSYERKEWQK--------

Wolsuc ----------------------EGAMQKIARYMNKEAHKAASLAFWQGDL----------

Burpse ------------------PAFFKIQLEKLVPLHGHARIRKWHAMLDEGRRGEL-------

Cloper --------------FENVNEQLDEALGVLKKHISQERVEQYKDLVAKGDYKKV-------

Despsy -----------------VRSKALAVVKSLRQRLGGKIVDQLSLLLQRDDLS---------

Desvul ---------GE-------TEAMLHGLERIRKRLGDDAWRRCADALRAG-----------D

Porgin -----------------DAEALANSFEHIARRMGGAATTQAITALKEGRLEE--------

Geosul ---------------PEYRDGMAGALERIRKKLGGDNYEAIKGHLDRWEMEP--------

Bdebac PRCAEENDILRAQALQLFARY-RQSLLAIQRKLGGLRAQEVMADLEKAELAYLNHAELSG

Niteur ASTVETREILRQQARQLF-DKYAGALQAISKKLGGDRFQEVSEDLENARSDFENKNEIQS

Borbro ------ALHGEQDGFGLYAARLRQSLDNIARRLGGERHRRLAALMDEALARQAGDGSVDA

Pseaer ------RVVGVEEGFAAFSAYLQKSLAGIVKRLGGERYQRLAAILVQALEEQGRDGSVDT

Pseput ------AVHGEEDGPRLFAERMLQSMANIYKRLGGERHQRLSEMLREALQEQQRSGAVDL

Psesyr ------SVKGEELGFGLFADRLLQSLNNIHKRLGGERHQRLLALMQTALEEQQRSGAVEL

Phopro ------AQNGEQQGWELFTEYLERGMFGIRKRLGMKRYEELLAAQKQAIATQKSNGTLAE

Sheone ------ERLGIEAGFNAFSQYLLQSLTSIRKRLGGKQYQELQDTMQQALSQQLNQNQTSQ

Esccol ------HAYGDEQGWQEYCEYLHHGLSAIKRRLGLQRYNELAARLDAALTTQLTTGSTDG

Shifle ------HAYGDEQGWQEYCEYLHHGLSAIKRRLGLQRYNELAAQLDTALTTQLTTGSTDG

Saltyp ------HAYGDGAGWQAYSEYLHHGLFAIRRRLGLQRFAELTDTLDRALAEQLSSGSTDG

. : .

Camjej ----LIVMLLEYYDKTYKKPDK---IDLELNTDDILKAKE---------ELLDHFKVQLL

Wolsuc -RRCAELLLVEYYDKVYKMEPCEYTILHTSKESTLKELERI------KGEILSSVRE---

Burpse ----FEELVDVHYDPAYTRSSGAHFARLPQAERFAFRPTAR-DVVEQARALLAHLDAAGA

Cloper ----ALELMEKYYDPRYGTSA------KKKTFRNELEINDIENELFKLEEIYKEISEELR

Despsy --EFVHILLTDYYDGRYGNCMLEYNYDLRLSSENI---------DDTVAELVKYRQSLL-

Desvul HTTAVR-ILLRYYDKAYAHHQAQQ--PREAVRHIVLERDDPATVARRLAADEDSLAG---

Porgin ----AVSLALDYYDKAYAHSLAEF--RESSSARLAVETDTPHETAIKLLEL-SHIKGLQS

Geosul ---FMEGLIRHYYDKLYYKTRDWVEDAVISLED--FSSGER--------ELNTFLASRRQ

Bdebac NKVWIEKLLQYYYDPMYLGSLE------RRHVTVLFKGTRF--------EAEKFVHSLT-

Niteur NKIWIEKLVRYYYDPLYLGSLQ------RRRVNPCFKGSGQ--------AVMDYLQARK-

Borbro HRAWIEPLLTEYYDPMYAYQRQ------AKAARIVFTGDHQ--------EVLDYLRGPGR

Pseaer HRGWIEGLLKEYYDPMYAFQRQ------SKEDRVEFRGNQA--------EVIGYLRQRQA

Pseput HRGWIEGLLNEYYDPMYAYQRA------AKAERIEFAGDAV--------EVREYLKARAL

Psesyr HRGWIEGLLGEYYDPMYAYQRE------HKAARIEFAGNQF--------EVSGYLSERSS

Phopro HDNWLRPLLIEYYDPMYTYQLS------KKADRIVFRGNYQ--------EVKSWLADK--

Sheone HLAWINLLLQKYYDPMYEYQLE------KKAHRVLFRGNHQ--------AMHEWLDNLSQ

Esccol HLAWLVPLLEEYYDPMYRYQLE------KKAEKVVFRGEWA--------EVAEWVKAR--

Shifle HLAWLVPLLKEYYDPMYRYQLE------KKAEKVVFRGEWA--------EVAVWVKAQ--

Saltyp HMAWLVPLLNEYYDPMYRYQLE------KKAANIVFRGTWQ--------DVANWLKAQ--

: :** *

Camjej F--------

Wolsuc ---------

Burpse RALDKRA--

Cloper EDVYEQ---

Despsy ---------

Desvul ---------

Porgin ---------

Geosul AAV------

Bdebac ---------

Niteur ---------

Borbro ALP------

Pseaer LRPS-----

Pseput RQPRK----

Psesyr RR-------

Phopro ---------

Sheone DSLSQENLG

Esccol ---------

Shifle ---------

Saltyp ---------
